# Small changes in ball position at address cause a chain effect in golf swing

**DOI:** 10.1038/s41598-020-79091-7

**Published:** 2021-01-29

**Authors:** Sung Eun Kim, Jangyun Lee, Sae Yong Lee, Hae-Dong Lee, Jae Kun Shim, Sung-Cheol Lee

**Affiliations:** 1 Department of Physical Education, Yonsei University, #321 Sports Science Complex, 50 Yonsei Ro, Seodaemun-gu, Seoul, 03722 Korea; 2 Frontier Research Institute of Convergence Sports Science, Yonsei University, Seoul, Korea; 3 Department of Orthopaedic Surgery, National Medical Center, Seoul, Korea; 4 Department of Orthopaedic Surgery, Seoul National University College of Medicine, Seoul, Korea; 5 Yonsei Institute of Sports Science and Exercise Medicine, Yonsei University, Seoul, Korea; 6 Department of Kinesiology, University of Maryland, 0110F School of Public Health (Bldg #255), 4200 Valley Drive, College Park, MD 20742 USA; 7 Neuroscience and Cognitive Science Program, University of Maryland, College Park, MD USA; 8 Maryland Robotics Center, University of Maryland, College Park, MD USA; 9 Department of Mechanical Engineering, Kyung Hee University, Yongin-si, Gyeonggi-do Korea

**Keywords:** Motor control, Sensory processing

## Abstract

The purpose of this study was to investigate how the ball position along the mediolateral (M-L) direction of a golfer causes a chain effect in the ground reaction force, body segment and joint angles, and whole-body centre of mass during the golf swing. Twenty professional golfers were asked to complete five straight shots for each 5 different ball positions along M-L: 4.27 cm (ball diameter), 2.14 cm (ball radius), 0 cm (reference position at preferred ball position), – 2.14 cm, and – 4.27 cm, while their ground reaction force and body segment motions were captured. The dependant variables were calculated at 14 swing events from address to impact, and the differences between the ball positions were evaluated using Statistical Parametric Mapping. The left-sided ball positions at address showed a greater weight distribution on the left foot with a more open shoulder angle compared to the reference ball position, whereas the trend was reversed for the right-sided ball positions. These trends disappeared during the backswing and reappeared during the downswing. The whole-body centre of mass was also located towards the target for the left-sided ball positions throughout the golf swing compared to the reference ball position, whereas the trend was reversed for the right-sided ball positions. We have concluded that initial ball position at address can cause a series of chain effects throughout the golf swing.

## Introduction

Biomechanics of golf swing has been a popular topic of research in the field of sport science not only because it is directly applicable to the golfer’s performance, but also because it can be used as a window to the underlying mechanisms of human movements realised through the parallel and serial connections of body segments. One of the main goals of golf swing is to maximise the club-head speed and accuracy, while taking advantage of force transfer^1,2^ through the kinematic chain of the body segments^3–5^. A large number of existing studies on golf swing has helped identify the kinematic and kinetic variables that are important for golf swing performance such as trunk rotation^6–8^, lower body flexion^9,10^, and centre of mass/pressure movement^11–14^, which have revealed important underlying mechanisms of golf swing in relation to its control. However, our knowledge regarding how the initial state of the human control system, such as the address position, influences the swing behaviour is largely limited. Due to the linked segments of the whole body used in golf swing, a small change in the address position can easily result in a chain effect^15–17^, serial events caused by an initial state, through the course of backswing and downswing followed by the address.

One of the important initial states in golf is the ball position relative to the golfer. Many golf coaches believe that the address position influences the entire golf swing causing a chain effect through the course of golf swing and eventually changes the outcome, a golf shot that eventually determines the distance, accuracy, and consistency. This is why coaches spend many hours on the address position of golfers to change and improve golf swing and shot^18,19^. ‘If they (golfers) are set incorrectly in posture, they can't work the body correctly… they're moving the wrong plane of movement, and then part of the reason why their club is moving in a funny fashion is because the body is actually moving incorrectly’, stated one coach. Another coach stated, ‘An incorrect posture at setup could have detrimental effects on the remainder of the swing. Any compensatory movement or counterbalances in the golf swing were results of poor posture’^19^. These coaches clearly understand that there is a chain effect in golf swing, which is influenced by the initial state at address.

Kim et al.^20^ studied the effect of the ball position along the mediolateral (M-L) direction, and found that the left-sided ball position causes open shoulder alignment at address as well as changes in club-head movement at impact. Another study by Bradshaw et al. investigated golfers with different handicap levels and found that the variability of the M-L ball position is related to the variability of the club velocity at impact^21^. Similarly, Zhang et al. found that the variability of the M-L ball position is related to the variability of the ball launch angle after impact^22^. Although these studies have revealed the importance of the ball position regarding the initial setup and final outcomes of golf swing, our knowledge on the influence of M-L ball position on the golfer’s movements during the course of golf swing is largely limited. A comprehensive study on the ball position and golf swing can potentially offer an opportunity to better understand the influences of the initial state on the consequences of swing behaviour. Furthermore, the outcomes of such a study can provide golf coaches and players with the knowledge of golf swing mechanisms in relation to the ball position and the insights into the improvement of coaching strategies, and eventually, golfer’s performance. For example, if a golf ball curves dramatically in flight from left to right for right-handed golfers (called ‘slice’ in golf), golf coaches and players often attribute it to insufficient shoulder rotation^23,24^, whereas a mal-positioned golf ball may be the cause of a chain effect: an open shoulder alignment^20^ leading to insufficient shoulder rotation^19^, and eventually, slice.

Contrary to other previous studies that focused more on the influence of M-L ball position on address position, club-head velocity at impact, and ball trajectory after impact, the current study investigates the behaviour of the golfer during the course of golf swing between address and impact. Our study examines the influence of the M-L ball position on the kinetic and kinematic variables considered to be critical in golf swing mechanism, such as ground reaction force (GRF), centre of pressure (COP), body segment and joint angles, and whole-body centre of mass (COM), during swing^1,25^. We hypothesise that a small change in ball position in the M-L direction at address will cause a chain effect through the course of the golf swing.

## Methods

### Participants

Twenty professional male golfers (mean ± standard deviation: age; 29.25 ± 4.20 years, height; 1.78 ± 0.06 m, mass; 77.96 ± 10.48 kg) volunteered to participate in this study. The participants had no history of chronic pain, major injuries, or surgery in at least the last 6 months. All procedures were approved and performed in accordance with the ethical standards of the Yonsei University institutional review board (IRB#1040917-201601-SB-104-02). Written informed consent was obtained from the participants before the experiment.

### Instrumentation

Testing was performed in an indoor facility using a motion capture system equipped with 8 infrared cameras (MX-F20; Vicon, Oxford Metrics, Oxford, UK; 250 Hz) that were synchronised with two force platforms (OR6-7; AMTI, Advanced Mechanical Technology, Inc., Watertown, MA, USA; 2000 Hz).

### Procedures

Thirty-five reflective markers with a 14-mm diameter were positioned on anatomical landmarks, based on the Vicon Plug-in-Gait full-body model (Oxford Metrics, Oxford, UK). Additionally, four reflective adhesive tapes were attached to the five-iron golf club (1 tape on the toe of the club head, 1 tape on the hosel, and 2 tapes on the shaft). Reflective adhesive tape was used on the golf ball.

**Figure 1:**
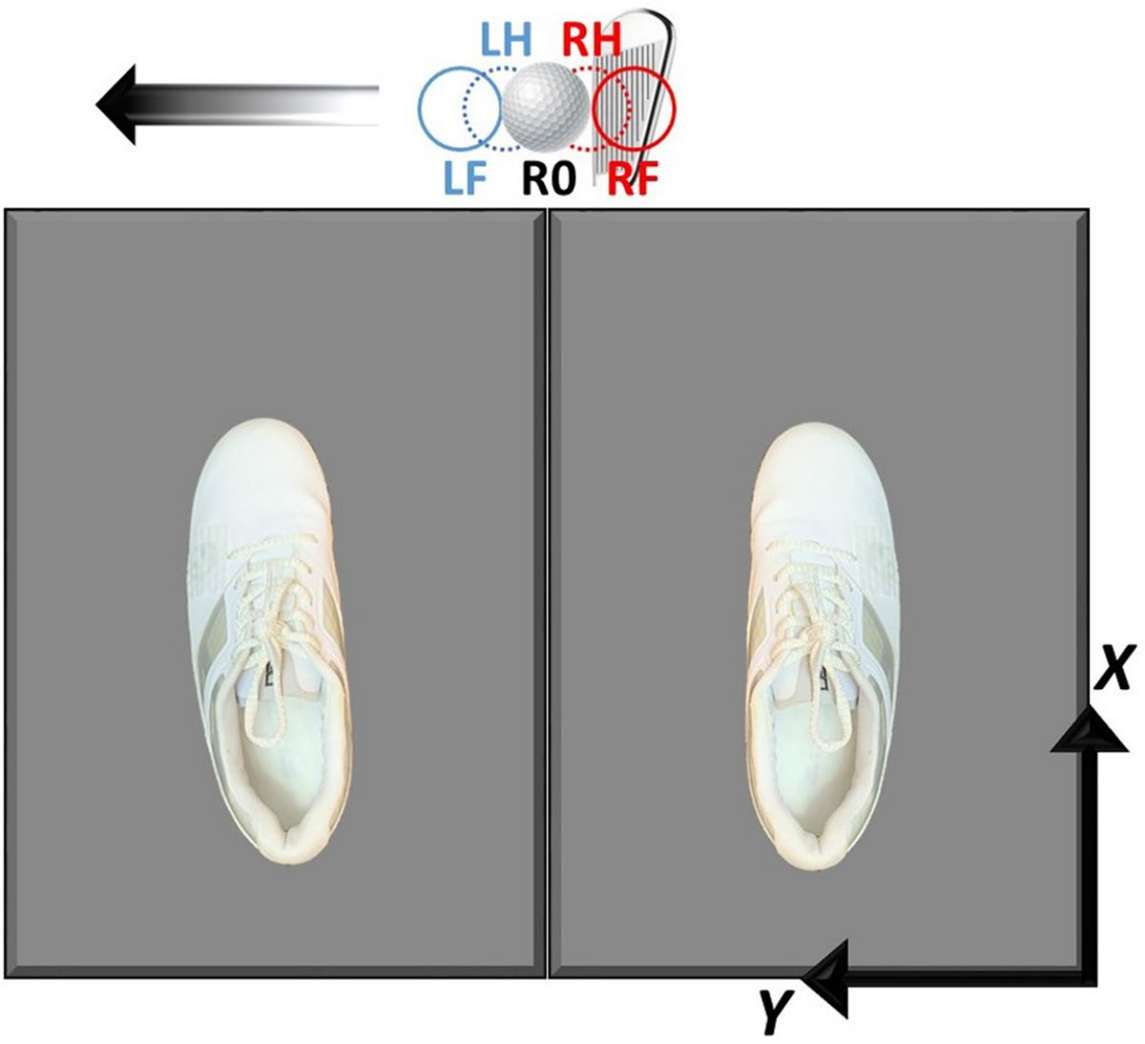
Participants were standing on 2 square force platforms and executed 5 shots for 5 different ball positions along mediolateral direction. LF, LH, R0, RH, and RF stand for left-full position (4.27 cm left from the preferred ball position), left-half position (2.14 cm left from the preferred ball position), reference ball position (i.e. 0 cm at the preferred ball position or the reference ball position), right-half position (2.14 cm right from the preferred ball position), and right-full position (4.27 cm right from the preferred ball position), respectively. The positive *Z* direction follows the right-hand-thumb rule.

The participants completed a self-selected warm-up with stretching and several golf shots for a minimum of 10 min. They performed an initial static, a standing calibration trial. Each participant was then asked to assume their preferred address position, and the ball position was marked invisible to them while the outlines of each foot was visibly drawn on the force platforms for consistent foot positions over multiple swings. The participants were asked to perform a straight shot that they would usually hit on a golf course towards a net 5 m away from them consistently over multiple trials. The participants performed five trials for each of five different ball positions along the M-L direction: 4.27 cm (left from the reference position) (LF), 2.14 cm (LH), 0 cm (reference position), – 2.14 cm (RH), and – 4.27 cm (RF) (Fig. 1). For each trial, the ball position was changed randomly and blindly. The diameter and radius of the golf ball were chosen as 4.27 cm and 2.14 cm, respectively, to displace the ball from the reference position. Thus, each participant performed 25 trials in total, five trials at each ball position. Additional shots were given to participants when they could not complete the full swing (2% of all shots) to complete five shots for each ball position condition. The laboratory coordinate system was set such that the *X* was anteroposterior (A–P) axis, *Y* was M-L axis, and *Z* was vertical axis (Fig. 1) following the right-hand-thumb rule.

### Data analysis

**Figure 2:**
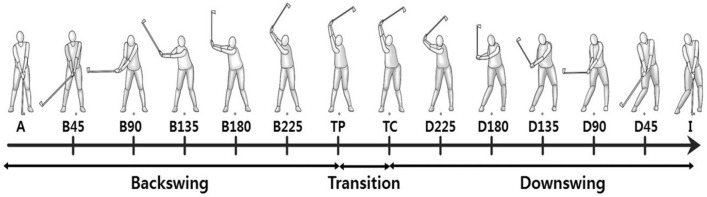
Fourteen sequential swing events identified in this study including address, 5 events during backswing, 2 events during transition to downswing, 5 events during downswing, and impact: Address (A), backswing 45° (B45), backswing 90° (B90), backswing 135° (B135), backswing 180° (B180), backswing 225° (B225), transition of the pelvis (TP), transition of the club (TC), downswing 225° (D225), downswing 180° (D180), downswing 135° (D135), downswing 90° (D90), downswing 45° (D45), and impact (I).

The three-dimensional data were smoothed using the Woltring filtering routine with a mean square error of 10 mm^2^^26^. The full swing was divided into 14 swing events using the club shaft angle and body moments. The 14 swing events were address (A), backswing 45° (B45), backswing 90° (B90), backswing 135° (B135), backswing 180° (B180), backswing 225° (B225), transition of pelvis (TP), transition of club (TC), downswing 225° (D225), downswing 180° (D180), downswing 135° (D135), downswing 90° (D90), downswing 45° (D45), and impact (I) (Fig. 2).

The address was defined as the frame immediately before the club initiates its movement away from the ball. In previous studies, the time when the club-head changes its direction along the mediolateral direction was identified as the transition from backswing to downswing (i.e. TC)^22,27,28^. However, the lower extremities typically start downswing actions before the change in club-head direction. To capture the initiation of downswing by the lower extremities, we added one more event, TP, which was identified as the frame when the pelvis changed its rotational direction. Impact was defined as the frame at which the club makes contact with the ball. The other events (B45, B90, B135, B180, B225, D225, D180, D135, D90, and D45) were identified using the angle of the club shaft in the *YZ* plane. Seventeen dependent variables were calculated at each of the 14 swing events as shown in Table 1. The following directions were considered as positive following the coordinate system shown in Fig. 1: vertical GRF, left foot lateral GRF, right foot medial GRF, anterior GRF, towards the target COP, anterior COP, open shoulder/pelvis angle in the transverse plane (left of the parallel to the target), trunk flexion angle in the sagittal plane, knee/ankle extension angle in the sagittal plane, towards the target COM, and anterior COM.

**Table 1:** List of kinematic and kinetic variables and descriptions.

Symbol	Variable name	Description
*GRF*_*Z,L*_ & *GRF*_*Z,R*_	Left & right vertical GRF	Ground reaction force along the *Z*-axis on left & right foot (+ : vertical)
*GRF*_*Y,L*_ & *GRF*_*Y,R*_	Left & right mediolateral GRF	Ground reaction force along the *Y*-axis on left (+ : lateral) & right foot (+ : medial)
*GRF*_*X,L*_ & *GRF*_*X,R*_	Left & right anteroposterior GRF	Ground reaction force along the *X*-axis on left & right foot (+ : anterior)
*COP*_*Y*_ & *COP*_*X*_	Mediolateral & anteroposterior COP	Centre of pressure on the force plates along the *Y-*axis & *X-*axis^11,12^ (+ : towards the target & anterior, respectively)
*A*_*S*_	Shoulder angle	Shoulder angle in the *XY* plane (i.e. transverse plane), where the shoulder was defined as a line between the right and left acromion markers^9,35,39,42,49–52^ (+ : open)
*A*_*P*_	Pelvis angle	Pelvis angle in the *XY* plane, where the pelvis was defined a line between the right and left anterior superior iliac spine markers^9,35,39,42,49–52^ (+ : open)
*A*_*T*_	Trunk angle	Trunk angle in the *XZ* plane (i.e. sagittal plane), where the trunk was defined as a line between the 7th cervical vertebrae and the mid-point of the right and left posterior superior iliac spine markers^53,54^ (+ : flexion)
*A*_*K.L*_ & *A*_*K,R*_	Left & right knee angle	Left & right knee joint angles between the thigh and shank in the *XZ* plane (+ : extension)
*A*_*A,L*_ & *A*_*A,R*_	Left & right ankle angle	Left & right ankle joint angles between the shank and foot in the *XZ* plane (+ : extension)
*COM*_*Y*_ & *COM*_*X*_	Mediolateral & anteroposterior COM	Whole-body centre of mass position along the *Y-*axis & *X-*axis (+ : towards the target & anterior, respectively)

### Statistical analysis

To capture the average behaviour throughout the golf swing, we calculated the average over all five trials for each condition under each dependent variable. For statistical analysis, Statistical Parametric Mapping (SPM)^29–32^ based on a random field theory was used to test statistical differences between ball positions using the open source (http://www.spm1d.org) MATLAB (Mathworks Inc., Natick, USA) code. SPM allows for non-directed hypothesis test and helps to control Type 1 error by reducing the number of statistical testing of one-dimensional data (i.e. 14 continuous swing events in our study). More details about the method can be found elsewhere^33,34^. We performed SPM on each dependent variable between the address and the impact. The critical significance was set at p < 0.01 instead of a more traditional p < 0.05 to partially compensate for the inflation of statistical error associated with multiple variables as suggested in other previous golf studies^11,27,35^.

## Results

**Figure 3:**
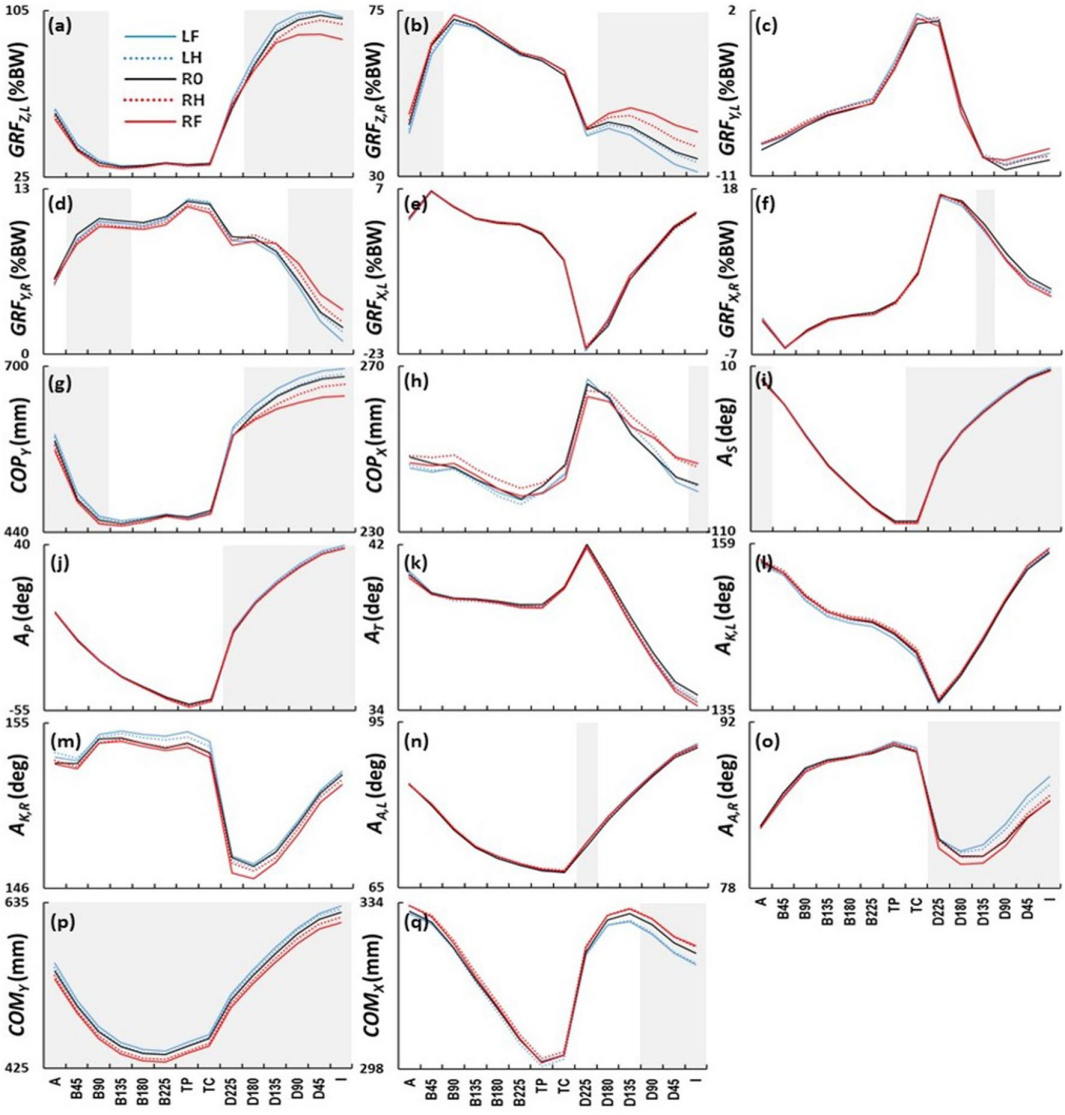
SPM results of the effect of mediolateral ball position on kinematic and kinetic variables during golf swing. LF, LH, R0, RH, and RF stand for left-full position (4.27 cm left from the reference), left-half position (2.14 cm left from the reference), reference ball position (i.e. preferred ball position), right-half position (2.14 cm right from the reference), and right-full position (4.27 cm right from the reference), respectively. The graphs show the mean trajectories across participants for **(a)** left foot vertical GRF (*GRF*_*Z,L*_) (+ : vertical), **(b)** right foot vertical GRF (*GRF*_*Z,R*_) (+ : vertical), **(c)** left foot mediolateral GRF (*GRF*_*Y,L*_) (+ : lateral), **(d)** right foot mediolateral GRF (*GRF*_*Y,R*_) (+ : medial), **(e)** left foot anteroposterior GRF (*GRF*_*X,L*_) (+ : anterior), **(f)** right foot anteroposterior GRF (*GRF*_*X,R*_) (+ : anterior), **(g)** mediolateral centre of pressure (*COP*_*Y*_) (+ : towards the target), **(h)** anteroposterior centre of pressure (*COP*_*X*_) (+ : anterior), **(i)** shoulder angle (*A*_*S*_) (+ : open), **(j)** pelvis angle (*A*_*P*_) (+ : open), **(k)** trunk angle (*A*_*T*_) (+ : flexion), **(l)** left knee angle (*A*_*K.L*_) (+ : extension), (m) right knee angle (*A*_*K,R*_) (+ : extension), **(n)** left ankle angle (*A*_*A,L*_) (+ : extension), **(o)** right ankle angle (*A*_*A,R*_) (+ : extension), **(p)** mediolateral centre of mass (*COM*_*Y*_) (+ : towards the target), and **(q)** anteroposterior centre of mass (*COM*_*X*_) (+ : anterior). The grey area indicates a significant difference between ball positions. The horizontal axes show 14 swing events (see Fig. 2). *%BW* percentage of body weight.

SPM analysis showed that the ball position was associated with systematic changes of *GRF*_*Z,L*_, *GRF*_*Z,R*_, *GRF*_*Y,R*_, *GRF*_*X,R*_, *COP*_*Y*_, *COP*_*X*_, *A*_*S*_, *A*_*P*_, *A*_*A,L*_, *A*_*A,R*_, *COM*_*Y*_, and *COM*_*X*_ (p < 0.01), whereas *GRF*_*Y,L*_, *GRF*_*X,L*_*,* *A*_*T*_*,* *A*_*K.L*_*,* and *A*_*K,R*_*,* did not show statistical differences between ball positions (Fig. 3). The results are presented below in the order of GRF variables, body segment and joint angles, and whole-body actions. More detailed mean ± SD for each variable at each event can be found online in the supplementary Table S1–S17.

In terms of the vertical GRF, *GRF*_*Z,L*_ showed that the left-sided ball positions were associated with greater *GRF*_*Z,L*_ magnitudes (LF: + 1.8%BW and LH: + 1.0%BW), whereas the right-sided ball positions were associated with smaller *GRF*_*Z,L*_ magnitudes (RH: – 1.1%BW, and RF: – 1.6%BW) between address and early backswing (A, B45, and B90) compared to R0 (42.0 ± 4.2%BW averaged across events then subjects) (p < 0.01) (Fig. 3a). The opposite trends were found in *GRF*_*Z,R*_ between address and early backswing (A and B45) (Fig. 3b), which showed smaller and greater magnitudes (LF: – 2.3%BW, LH: – 1.3%BW, RH: + 1.1%BW, and RF: + 1.6%BW) in the left-sided and right-sided ball positions, respectively, compared to R0 (60.7 ± 4.7%BW) (p < 0.01). These trends disappeared after B90 and B45, respectively, and reappeared during the events between downswing and impact (D180, D135, D90, D45, and I) for both *GRF*_*Z,L*_ (LF: + 2.6%BW, LH: + 1.3%BW, RH: – 2.6%BW, and RF: – 6.6%BW) compared to R0 (95.4 ± 10.0%BW) (p < 0.01) and *GRF*_*Z,R*_ (LF: – 2.8%BW, LH: – 0.7%BW, RH: + 2.9%BW, and RF: + 5.7%BW) compared to R0 (40.0 ± 10.8%BW) (p < 0.01) (Fig. 3a,b). In terms of the right foot mediolateral GRF, *GRF*_*Y,R*_ showed smaller magnitudes (less towards the target) (LF: – 0.3%BW, LH: – 0.3%BW RH: – 0.5%BW, and RF: – 0.6%BW) for both left-sided and right-sided ball positions during early backswing (B45, B90, and B135) as compared to R0 (+ 10.2 ± 2.3%BW) (p < 0.01) (Fig. 3d). This trend disappeared during backswing and reappeared during late downswing and impact (D90, D45, and I), but a different trend reappeared, smaller and greater magnitudes (less and more towards the target) (LF: – 0.7%BW, LH: – 0.1%BW, RH: + 0.6%BW, and RF: + 1.4%BW) in the left-sided and right-sided ball positions, respectively, compared to R0 (+ 3.7 ± 4.0%BW) (p < 0.01) (Fig. 3d). In terms of the right foot anteroposterior GRF, *GRF*_*X,R*_ showed smaller magnitudes (less anterior direction) (LF: – 1.0%BW, LH: – 1.0%BW, RH: – 0.7%BW, and RF: – 0.7%BW) for both left-sided and right-sided ball positions at D135 compared to R0 (+ 12.6 ± 3.3%BW across subjects) (p < 0.01) (Fig. 3f). Regarding the mediolateral COP, *COP*_*Y*_ showed trends towards and distant to the target position values (LF: + 9.0 mm, LH: + 5.1 mm, RH: – 5.1 mm, and RF: – 7.9 mm), respectively, in the left-sided and right-sided ball positions between address and early backswing (A, B45, and B90) compared to R0 (+ 510.9 ± 27.5 mm) (p < 0.01) (Fig. 3g). This trend disappeared after B90; however, the same trend (LF: + 12.1 mm, LH: + 3.2 mm, RH: – 11.4 mm, and RF: – 23 mm) compared to R0 (+ 662.7 ± 42.2 mm), reappeared at the events between downswing and impact (D180, D135, D90, D45, and I) (p < 0.01) (Fig. 3g). In terms of the anteroposterior COP, *COP*_*X*_ showed trends towards the posterior and anterior direction position values (LF: – 1.7 mm, LH: – 0.5 mm, RH: + 4.3 mm, and RF: + 5.2 mm), respectively, in the left-sided and right-sided ball positions at impact (I) compared to R0 (+ 241.4 ± 43.3 mm) (p < 0.01) (Fig. 3h).

*A*_*S*_ showed that the left-sided ball positions were generally associated with open shoulder angles (LF: + 0.5° and LH: – 0.1°) at address, the right-sided ball positions were associated with the closed shoulder angles (RH: – 0.6° and RF: – 1.0°) compared to R0 (+ 0.2° ± 3.0°) (p < 0.01) (Fig. 3i). This trend disappeared during backswing. However, the same trend, open and closed shoulder angles (LF: + 1.3°, LH: + 0.7°, RH: – 0.3°, and RF: – 0.5°), respectively, in the left-sided and right-sided ball positions reappeared between downswing and impact (TC, D225, D180, D135, D90, D45, and I) compared to R0 (– 32.3 ± 6.3°) (p < 0.01) (Fig. 3i). *A*_*P*_ also showed that the left-sided ball positions were generally associated with open pelvis (LF: + 1.6° and LH: + 0.8°) between downswing and impact (D225, D180, D135, D90, D45, and I) as compared to R0 (+ 19.1 ± 9.3°), whereas the right-sided ball position was associated with the closed pelvis angle (RH: + 0.1° and RF: – 0.2°) at the events between late downswing and impact (D135, D90, D45, and I) compared to R0 (+ 29.5 ± 9.5°) (p < 0.01) (Fig. 3j). *A*_*A,L*_ showed more flexed left ankle angles (LF: + 0.7°, LH: + 0.7°, RH: + 0.6°, and RF: + 0.7°) for both left-sided and right-sided ball positions at D225 compared to R0 (+ 72.5 ± 6.3°) (p < 0.01) (Fig. 3n). *A*_*A,R*_ showed generally more extend and flexed right ankle angles (LF: + 1.1°, LH: + 0.7° RH: + 0.1°, and RF: – 0.4°) between downswing and impact (D225, D180, D135, D90, D45, and I) compared to R0 (+ 82.5 ± 6.3°) (p < 0.01) (Fig. 3o).

In terms of the COM, *COM*_*Y*_ showed trends towards and distant to the target position values (LF: + 6.7 mm, LH: + 3.0 mm, RH: – 5.7 mm, RF: – 10.1 mm) in the left-sided and right-sided ball positions, respectively, throughout the whole swing between address and impact compared to R0 (+ 516.4 ± 36.5 mm) (p < 0.01) (Fig. 3p). In contrast, *COM*_*X*_ showed trends towards the posterior and anterior direction position values (LF: – 2.3 mm, LH: – 2.0 mm, RH: + 1.3 mm, and RF: + 1.4 mm) in the left-sided and right-sided ball positions, respectively, between late downswing and impact (D90, D45, and I) compared to R0 (+ 325.9 ± 29.1 mm) (p < 0.01) (Fig. 3q).

## Discussion

We examined the effects of M-L ball position on GRF, body segment and joint angles, and whole-body actions during golf swing, and we found that the M-L ball position systematically influenced the *GRF*_*Z,L*_, *GRF*_*Z,R*_, *GRF*_*Y,R*_, *GRF*_*X,R*_, *COP*_*Y*_, *COP*_*X*_, *A*_*S*_, *A*_*P*_, *A*_*A,L*_, *A*_*A,R*_, *COM*_*Y*_, and *COM*_*X*_ (Fig. 3).

### At address

**Figure 4:**
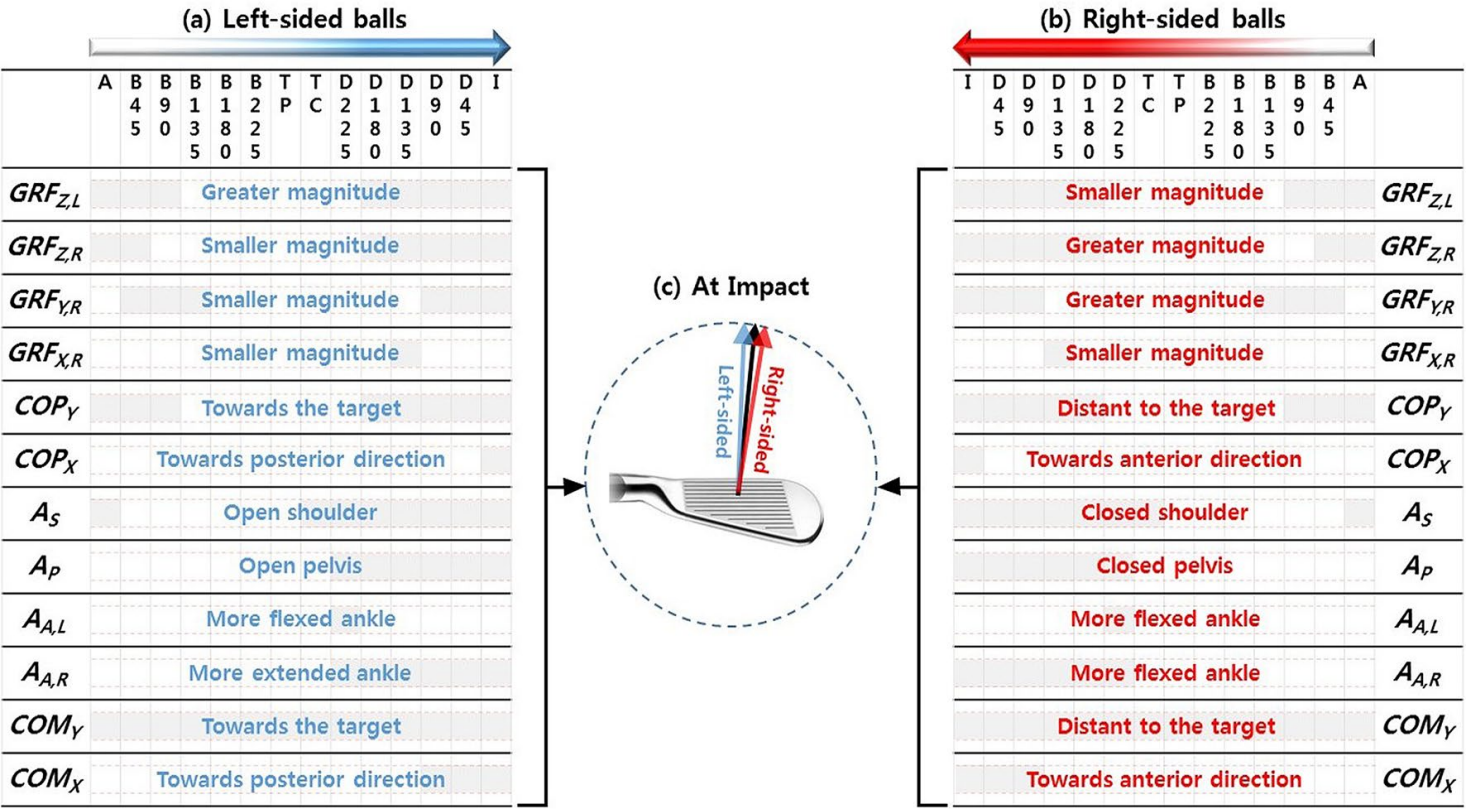
Summary of changes in golf swing due to changes in ball position for GRF variables, body segment and joint angles, and whole-body actions. These variables that showed statistical significance in SPM are shown in **(a,b)**. **(a)** Changes in kinematic and kinetic variables in the left-sided ball positions across swing events from address (A) to impact (I), **(b)** changes in kinematic and kinetic variables in the right-sided ball positions, and **(c)** club path at impact (refers to Kim et al.’s previous study). The general trends are described in **(a,b)**. The grey areas show the parts with statistical significance (p < .01). *GRF*_*Z,L*_ left foot vertical GRF, *GRF*_*Z,R*_ right foot vertical GRF, *GRF*_*Y,R*_ right foot mediolateral GRF, *GRF*_*X,R*_ right foot anteroposterior GRF, *COP*_*Y*_ mediolateral centre of pressure, *COP*_*X*_ anteroposterior centre of pressure, *A*_*S*_ shoulder angle, *A*_*P*_ pelvis angle, *A*_*A,L*_ left ankle angle, *A*_*A.R*_ right ankle angle, *COM*_*Y*_ mediolateral centre of mass, *COM*_*X*_ anteroposterior centre of mass.

Our findings on the differences between ball positions at address are consistent with a previous study^20^. Kim et al*.* (2018) showed that the left-sided ball position was associated with a greater/smaller vertical GRF on the left/right foot, and a more open position of shoulder segment. In a quasi-static posture at address, the whole-body position demonstrated by COP and COM in the M-L direction can be predicted from the difference in the vertical GRFs of feet (*GRF*_*Z,L*_ and *GRF*_*Z,R*_). Consistent with the mechanics, our study reports that the whole-body position demonstrated by *COP*_*Y*_ and *COM*_*Y*_ was towards/distant to the target in the M-L direction (Fig. 4a,b). We also found that the shoulder angular position (*A*_*S*_) showed systematic differences between the ball position at address. The shoulder in the left-sided ball position was more open than that at the reference ball position (Fig. 4a), whereas the trend was reversed in the right-sided ball position (Fig. 4b).

### During backswing

Our study shows that the trends GRF and COP observed at address continues during early backswing, which suggests that the initial setup (*GRF*_*Z,L*_, *GRF*_*Z,R*_, and *COP*_*Y*_) at address caused by the ball position continued during early backswing, demonstrating a chain effect (Fig. 4a,b). The mediolateral GRF on the right foot (*GRF*_*Y,R*_) showed a difference between the ball positions during early backswing, where the vertical GRF on the right foot (*GRF*_*Z,R*_) generally showed peak magnitude. The mediolateral GRF magnitude at the right foot was smaller (less towards the target) for both left-sided and right-sided ball positions than at the reference ball position during early backswing. The trend COM in the M-L direction (*COM*_*Y*_) observed at address continued throughout the whole backswing, which also demonstrated a chain effect (Fig. 4a,b).

### During back-to-downswing transition

The difference between the shoulder angles (*A*_*S*_) of the ball positions observed at address reappeared during back-to-downswing transition (TC). However, the shoulder angle during the transition phase showed more closed positions for both the left-sided and right-sided ball positions compared to the reference ball position. The participants rotated their shoulder more and created a closed shoulder position during the transition for the left-sided ball positions. This seemed to be a strategy employed by the participants to better utilise the X-factor^7,36–38^ to absorb greater rotational potential energy during the transition to hit the ball at a longer distance from the club-head at TC when the ball is positioned left. We also found that COM in the M-L direction (*COM*_*Y*_) observed at address and backswing continued during back-to-downswing transition (Fig. 4a,b), suggesting a chain effect.

### During downswing

Many of the kinematic and kinetic variables showed differences during downswing (*GRF*_*X,R*_*,* *A*_*P*_, *A*_*A,L*_, *A*_*A,R*_, *COM*_*Y*_, and *COM*_*X*_), whereas the differences between the ball positions observed at address or during early backswing reappeared during downswing (*GRF*_*Z,L*_, *GRF*_*Z,R*_, *GRF*_*Y,R*_*,* *COP*_*Y*_, *A*_*S*_).

The systematic differences between GRF and COP (*GRF*_*Z,L*_, *GRF*_*Z,R*_, and *COP*_*Y*_) of the ball positions observed at address and during early backswing reappeared during mid-downswing and continued throughout the impact (Fig. 4a,b). The difference in mediolateral GRF on the right foot (*GRF*_*Y,R*_) observed during early backswing also reappeared during the later downswing. However, the mediolateral GRF on the right foot during the later downswing showed smaller/greater magnitude (less/more towards the target) in the left-/right-sided ball positions than that at the reference ball position (Fig. 4a,b). The anteroposterior GRF on the right foot (*GRF*_*X,R*_) at the mid-downswing showed smaller magnitude (less anterior direction) for both the left-sided and right-sided ball positions compared to the reference ball position (Fig. 4a,b).

The difference between the shoulder angles (*A*_*S*_) of the ball positions observed at back-to-downswing transition continued throughout the downswing. However, the shoulder angle during the early downswing showed a more open position for both the left-sided and right-sided ball positions compared to the reference ball position, and the same trend was observed for the pelvis angle (*A*_*P*_). The participants rotated their shoulder and pelvis more and created open shoulder and pelvis positions during the early downswing for the right-sided ball position potentially to hit the ball at a shorter distance from the club-head at TC. However, we found that the systematic difference in shoulder angle observed at address reappeared during the mid-downswing, and the same trend was observed in pelvis angle (Fig. 4a,b).

The golf swing during the downswing is executed through linear transition and angular rotation of the body^6,11,12,39–41^. The former can be estimated with the COP in the M-L direction (*COP*_*Y*_), and the latter with the shoulder and pelvis angles (*A*_*S*_ and *A*_*P*_). COP in the M-L direction during the downswing and impact showed a greater difference in the right-sided ball positions (*RF*: – 23 mm) than that in the left-sided ball positions (*LF*: + 12.1 mm), whereas the pelvis angle during the downswing and impact showed a smaller difference in the right-sided ball position (*RF*: – 0.2°) than that in the left-sided ball positions (*LF*: + 1.6°), which demonstrates that the proportion of the linear transition of the body is greater in percentage than the angular rotation of the pelvis in the right-sided ball positions.

Furthermore, the left ankle angle (*A*_*A,L*_) was more flexed for both the left-sided and right-sided ball positions compared to the reference ball position during the early downswing (Fig. 4a,b). The right ankle angle (*A*_*A,R*_) was less/more flexed for the left-/right-sided ball positions compared to the reference ball position throughout the whole downswing (Fig. 4a,b).

COM in the M-L direction (*COM*_*Y*_) shows that the whole-body position is more toward/distant to the target in the left-/right-sided ball positions throughout the whole swing (Fig. 4a,b). This observation also confirms that the chain effect of the ball position continuously leads to systematic changes in the final stage of golf swing. COM in the A-P direction (*COM*_*X*_) was in a more posterior/anterior position compared to the reference ball position in the left-/right-sided ball positions during late downswing (Fig. 4a,b).

### At impact

Most of the kinematic and kinetic variables analysed in our study showed differences during downswing continues at impact (*GRF*_*Z,L*_, *GRF*_*Z,R*_, *GRF*_*Y,R*_*,* *COP*_*Y*_, *A*_*S*_, *A*_*P*_, *A*_*A,R*_, *COM*_*Y*_, and *COM*_*X*_), demonstrating the chain effect (Fig. 4a,b). We also found that the anteroposterior COP (*COP*_*X*_) was in a more posterior/anterior position compared to the reference ball position in the left-/right-sided ball positions at impact (Fig. 4a,b).

Although our study did not directly investigate club-head kinematics or club-ball interaction at impact since the focus was on the swing action influenced by the ball position, another study by Kim et al*.* (2018) analysed the effect of the M-L ball position on the club-head kinematics and showed that the club path had less/more ‘in–out’ trajectory at impact in the left-/right-sided ball positions^20^. In our study, the shoulder angle (*A*_*S*_) is more open during downswing and at impact in the left-sided ball positions. The less ‘in–out’ trajectory of the club-head at impact in the left-sided ball positions reported in the previous study^20^ may be due to a more open shoulder angle during the downswing and impact, whereas the increased ‘in–out’ in the right-sided ball positions at impact may be due to a more closed shoulder angle during the downswing and impact (Fig. 4c), demonstrating another chain effect.

### Statistics and practical applications

Previous studies on golf swing often chose limited discrete swing events for analysis such as address, swing transition, mid-downswing, impact, or the time of the maximum/minimum dependent variable^28,35,41–43^. However, our study employed SPM recently introduced in biomechanics^29–33,44^ to analyse the entire golf swing at different discrete swing events. Since golfers use different swing tempos^22,45^, and swing events between golfers do not usually occur at the same time in the time trajectory of swing, it was not ideal to apply SPM to the time trajectory. Therefore, we identified and used 14 sequential swing events for SPM analysis to allow the comparison of dependent variables across swing events rather than time trajectories.

Our findings can potentially be informative to golf coaches and golfers. For example, when a golfer shows a lack of shoulder and pelvis rotations during the downswing, the coach can check whether the ball position is too far to the right and move the ball to the left, and then assess whether the golfer demonstrates improved shoulder and pelvis rotation for the desired swing flow^6,39,46,47^. Thus, the findings of the current study may provide valuable knowledge to swing coaches and golfers to improve swing and eventually the performance.

### Limitations

The goal of the study was to investigate if there existed systematic differences in dependent variables at ball positions. Although some variables showed statistical differences at ball positions, the interpretation of the results in practical settings should consider the differences between statistical significance and practical significance. For example, the sizes of some of the effects are very small (e.g. ~ 1.3 mm differences in *COM*_*X*_ between conditions), particularly considering the accuracy of the motion capture system (~ 2 mm)^48^. Although these differences may be statistically significant, they may not carry a practical significance because the differences are larger than the accuracy of the motion capture system.

## Conclusion

The purpose of this study to investigate how the ball position influences golf swing in relation to the chain effect^15–17^. Our study found that the M-L ball position systematically influenced the weight distribution, shoulder and pelvis angles, left/right ankle angles, and whole-body action during the golf swing and demonstrated the chain effect. Based on the results of this study, we concluded that the chain effect exists in golf swing: the initial state of the address caused by the ball position can lead to sequential changes in golf swing behaviour.

## Supplementary Information

Supplementary Tables.

## Data Availability

Upon reasonable request, the datasets used and analysed during the current study will be made available by the corresponding author.
