# Supplementary material for: Small changes in ball position at address cause a chain effect in golf swing
Source: Sci Rep. 2021 Jan 29;11:2694. doi: 10.1038/s41598-020-79091-7 (PMC7846748; doi:10.1038/s41598-020-79091-7)
Supplement: Supplementary file 1 — Supplementary Tables. [file 41598_2020_79091_MOESM1_ESM.docx]

**Small changes in ball position at address cause a chain effect in golf swing**

**Sung Eun Kim^1,2^, Jangyun Lee^3,4^, Sae Yong Lee^1,5^, Hae-Dong Lee^1,2^, Jae Kun Shim^6,7,8,9^* & Sung-Cheol Lee^1,2^***

^1^Department of Physical Education, Yonsei University, Seoul, Korea

^2^Frontier Research Institute of Convergence Sports Science, Yonsei University, Seoul, Korea

^3^Department of Orthopaedic Surgery, National Medical Center, Seoul, Korea

^4^Department of Orthopaedic Surgery, Seoul National University College of Medicine, Seoul, Korea

^5^Yonsei Institute of Sports Science and Exercise Medicine, Yonsei University, Seoul, Korea

^6^Department of Kinesiology, University of Maryland, College Park, MD, USA

^7^Neuroscience and Cognitive Science Program, University of Maryland, College Park, MD, USA

^8^Maryland Robotics Center, University of Maryland, College Park, MD, USA

^9^Department of Mechanical Engineering, Kyung Hee University, Yongin-Si, Gyeonggi-do, Korea

***Corresponding Authors:**

**J.K.S.**

0110F School of Public Health (Bldg #255), 4200 Valley Drive, University of Maryland College Park, MD 20742, USA

Tel.: +1 (301) 405-2492; email: [jkshim@umd.edu](mailto:jkshim@umd.edu)

**S.C.L.**

#321 Sports Science Complex, 50 Yonsei Ro,

Seodaemun-gu, Seoul, 03722 Korea

Tel.: +82 (2) 2123-3192; email: [cheol3192@yonsei.ac.kr](mailto:cheol3192@yonsei.ac.kr)

**Table S1.** Left foot vertical ground reaction force (*GRF_Z,L_*) (mean ± SD) for 5 mediolateral ball positions over fourteen swing events analyzed: Address (A), backswing 45° (B45), backswing 90° (B90), backswing 135° (B135), backswing 180° (B180), backswing 225° (B225), transition of the pelvis (TP), transition of the club (TC), downswing 225° (D225), downswing 180° (D180), downswing 135° (D135), downswing 90° (D90), downswing 45° (D45), and impact (I). LF, LH, R0, RH, and RF stand for left-full position (4.27 cm left from the reference), left-half position (2.14 cm left from the reference), reference ball position (i.e. preferred ball position), right-half position (2.14 cm right from the reference), and right-full position (4.27 cm right from the reference), respectively. %BW: percentages of body weight.

|  | **LF** | **LH** | **R0** | **RH** | **RF** |
| --- | --- | --- | --- | --- | --- |
| A (%BW) | 57.8 ± 3.9 | 56.8 ± 5.0 | 55.6 ± 4.9 | 54.4 ± 4.6 | 52.8 ± 4.2 |
| B45 (%BW) | 40.6 ± 5.9 | 39.8 ± 6.0 | 38.6 ± 6.3 | 37.7 ± 6.3 | 37.7 ± 5.7 |
| B90 (%BW) | 32.9 ± 6.3 | 32.4 ± 6.6 | 31.8 ± 6.2 | 30.7 ± 6.2 | 30.6 ± 5.6 |
| B135 (%BW) | 30.6 ± 6.5 | 30.2 ± 6.3 | 30.0 ± 5.7 | 29.2 ± 5.7 | 29.1 ± 5.6 |
| B180 (%BW) | 30.8 ± 6.5 | 30.5 ± 6.3 | 30.6 ± 5.8 | 29.8 ± 6.2 | 29.9 ± 5.9 |
| B225 (%BW) | 31.9 ± 7.7 | 31.3 ± 7.6 | 31.8 ± 7.1 | 31.3 ± 7.6 | 31.5 ± 6.9 |
| TP (%BW) | 30.4 ± 8.6 | 30.1 ± 8.4 | 31.0 ± 7.9 | 30.4 ± 8.6 | 30.6 ± 7.6 |
| TC (%BW) | 30.7 ± 6.3 | 30.9 ± 6.4 | 31.5 ± 5.9 | 30.9 ± 6.9 | 30.8 ± 5.2 |
| D225 (%BW) | 61.9 ± 15.9 | 60.9 ± 14.8 | 57.8 ± 12.1 | 58.9 ± 15.8 | 60.0 ± 15.7 |
| D180 (%BW) | 82.1 ± 15.1 | 79.7 ± 15.2 | 78.1 ± 12.7 | 76.4 ± 15.6 | 76.3 ± 16.2 |
| D135 (%BW) | 98.0 ± 15.3 | 95.6 ± 18.5 | 94.4 ± 14.8 | 91.0 ± 18.5 | 89.4 ± 16.4 |
| D90 (%BW) | 103.4 ± 12.9 | 101.7 ± 15.4 | 100.6 ± 14.0 | 97.9 ± 16.7 | 93.4 ± 14.2 |
| D45 (%BW) | 104.6 ± 13.8 | 104.4 ± 14.8 | 102.8 ± 13.4 | 100.2±15.8 | 93.6 ± 12.0 |
| I (%BW) | 101.8 ± 15.4 | 101.8 ± 15.2 | 101.0 ± 13.1 | 98.3 ± 15.7 | 91.2 ± 13.4 |

**Table S2.** Right foot vertical ground reaction force (*GRF_Z,R_*) (mean ± SD) for 5 mediolateral ball positions over fourteen swing events analyzed: Address (A), backswing 45° (B45), backswing 90° (B90), backswing 135° (B135), backswing 180° (B180), backswing 225° (B225), transition of the pelvis (TP), transition of the club (TC), downswing 225° (D225), downswing 180° (D180), downswing 135° (D135), downswing 90° (D90), downswing 45° (D45), and impact (I). LF, LH, R0, RH, and RF stand for left-full position (4.27 cm left from the reference), left-half position (2.14 cm left from the reference), reference ball position (i.e. preferred ball position), right-half position (2.14 cm right from the reference), and right-full position (4.27 cm right from the reference), respectively. %BW: percentages of body weight.

|  | **LF** | **LH** | **R0** | **RH** | **RF** |
| --- | --- | --- | --- | --- | --- |
| A (%BW) | 41.8 ± 4.1 | 43.0 ± 5.3 | 44.1 ± 5.1 | 45.4 ± 4.8 | 46.9 ± 4.4 |
| B45 (%BW) | 63.1 ± 6.3 | 63.9 ± 6.7 | 65.4 ± 7.1 | 66.2 ± 7.3 | 65.8 ± 6.2 |
| B90 (%BW) | 71.5 ± 6.5 | 72.3 ± 6.7 | 72.6 ± 6.2 | 73.8 ± 6.3 | 73.8 ± 5.7 |
| B135 (%BW) | 70.3 ± 6.5 | 70.5 ± 6.1 | 70.7 ± 5.5 | 71.6 ± 5.7 | 71.7 ± 5.5 |
| B180 (%BW) | 66.6 ± 6.8 | 67.1 ± 6.0 | 67.0 ± 5.6 | 67.7 ± 6.2 | 67.7 ± 5.9 |
| B225 (%BW) | 62.8 ± 7.3 | 63.2 ± 6.7 | 63.2 ± 6.5 | 63.6 ± 7.0 | 63.6 ± 6.6 |
| TP (%BW) | 61.7 ± 7.1 | 62.2 ± 7.1 | 61.2 ± 6.9 | 62.0 ± 7.3 | 62.1 ± 5.8 |
| TC (%BW) | 58.6 ± 6.7 | 58.4 ± 6.0 | 57.5 ± 6.1 | 58.4 ± 6.9 | 58.7 ± 5.6 |
| D225 (%BW) | 41.0 ± 5.9 | 41.4 ± 6.5 | 42.6 ± 4.9 | 42.7 ± 6.4 | 43.1 ± 5.8 |
| D180 (%BW) | 43.0 ± 8.2 | 44.0 ± 8.6 | 44.7 ± 6.9 | 46.0 ± 7.9 | 47.1 ± 8.2 |
| D135 (%BW) | 41.1 ± 11.1 | 43.0 ± 11.3 | 43.5 ± 10.3 | 46.5 ± 10.9 | 48.6 ± 11.6 |
| D90 (%BW) | 37.1 ± 13.0 | 39.5 ± 13.4 | 40.0 ± 12.6 | 43.8 ± 13.6 | 46.9 ± 13.9 |
| D45 (%BW) | 33.3 ± 13.1 | 36.1 ± 13.8 | 36.7 ± 13.3 | 40.1 ± 14.0 | 44.0 ± 14.3 |
| I (%BW) | 31.2 ± 13.0 | 33.8 ± 13.6 | 34.8 ± 13.5 | 38.0 ± 13.9 | 42.0 ± 13.9 |

**Table S3.** Left foot mediolateral ground reaction force (*GRF_Y,L_*) (mean ± SD) for 5 mediolateral ball positions over fourteen swing events analyzed: Address (A), backswing 45° (B45), backswing 90° (B90), backswing 135° (B135), backswing 180° (B180), backswing 225° (B225), transition of the pelvis (TP), transition of the club (TC), downswing 225° (D225), downswing 180° (D180), downswing 135° (D135), downswing 90° (D90), downswing 45° (D45), and impact (I). LF, LH, R0, RH, and RF stand for left-full position (4.27 cm left from the reference), left-half position (2.14 cm left from the reference), reference ball position (i.e. preferred ball position), right-half position (2.14 cm right from the reference), and right-full position (4.27 cm right from the reference), respectively. %BW: percentages of body weight.

|  | **LF** | **LH** | **R0** | **RH** | **RF** |
| --- | --- | --- | --- | --- | --- |
| A (%BW) | –8.6 ± 1.8 | –8.4 ± 1.8 | –8.9 ± 2.1 | –8.4 ± 1.8 | –8.4 ± 1.8 |
| B45 (%BW) | –8.0 ± 2.8 | –7.9 ± 2.9 | –8.1 ± 3.0 | –7.7 ± 3.0 | –7.8 ± 2.9 |
| B90 (%BW) | –6.9 ± 2.8 | –6.9 ± 2.9 | –7.1 ± 2.7 | –6.7 ± 2.8 | –6.9 ± 2.8 |
| B135 (%BW) | –6.0 ± 2.5 | –6.0 ± 3.0 | –6.3 ± 2.4 | –5.9 ± 2.7 | –6.1 ± 2.7 |
| B180 (%BW) | –5.4 ± 2.6 | –5.5 ± 3.0 | –5.8 ± 2.5 | –5.5 ± 2.9 | –5.7 ± 2.6 |
| B225 (%BW) | –4.9 ± 3.0 | –5.0 ± 3.3 | –5.3 ± 2.8 | –5.1 ± 3.3 | –5.3 ± 3.0 |
| TP (%BW) | –2.0 ± 2.8 | –2.3 ± 2.9 | –2.5 ± 2.6 | –2.3 ± 2.9 | –2.6 ± 2.6 |
| TC (%BW) | 1.8 ± 3.1 | 1.2 ± 3.4 | 1.0 ± 3.0 | 1.3 ± 3.3 | 1.4 ± 3.4 |
| D225 (%BW) | 1.0 ± 4.8 | 1.3 ± 4.8 | 1.2 ± 4.7 | 1.4 ± 5.3 | 0.8 ± 5.5 |
| D180 (%BW) | –6.2 ± 4.9 | –5.6 ± 4.6 | –5.5 ± 4.3 | –5.4 ± 4.6 | –6.0 ± 5.3 |
| D135 (%BW) | –9.4 ± 3.9 | –9.3 ± 3.7 | –9.5 ± 3.4 | –9.6 ± 3.8 | –9.6 ± 4.1 |
| D90 (%BW) | –10.1 ± 3.1 | –9.9 ± 3.5 | –10.5 ± 3.0 | –10.2 ± 3.8 | –9.8 ± 3.2 |
| D45 (%BW) | –9.7 ± 2.8 | –9.6 ± 3.5 | –10.1 ± 3.4 | –9.7 ± 3.7 | –9.3 ± 3.2 |
| I (%BW) | –9.2 ± 2.7 | –9.5 ± 3.3 | –9.8 ± 3.3 | –9.4 ± 3.3 | –8.9 ± 3.0 |

**Table S4.** Right foot mediolateral ground reaction force (*GRF_Y,R_*) (mean ± SD) for 5 mediolateral ball positions over fourteen swing events analyzed: Address (A), backswing 45° (B45), backswing 90° (B90), backswing 135° (B135), backswing 180° (B180), backswing 225° (B225), transition of the pelvis (TP), transition of the club (TC), downswing 225° (D225), downswing 180° (D180), downswing 135° (D135), downswing 90° (D90), downswing 45° (D45), and impact (I). LF, LH, R0, RH, and RF stand for left-full position (4.27 cm left from the reference), left-half position (2.14 cm left from the reference), reference ball position (i.e. preferred ball position), right-half position (2.14 cm right from the reference), and right-full position (4.27 cm right from the reference), respectively. %BW: percentages of body weight.

|  | **LF** | **LH** | **R0** | **RH** | **RF** |
| --- | --- | --- | --- | --- | --- |
| A (%BW) | 5.6 ± 2.2 | 5.5 ± 2.1 | 6.0 ± 2.6 | 5.6 ± 2.2 | 5.9 ± 2.3 |
| B45 (%BW) | 9.0 ± 2.3 | 8.9 ± 2.3 | 9.4 ± 2.3 | 8.8 ± 2.4 | 8.6 ± 2.3 |
| B90 (%BW) | 10.5 ± 2.3 | 10.4 ± 2.4 | 10.7 ± 2.4 | 10.2 ± 2.5 | 10.1 ± 2.4 |
| B135 (%BW) | 10.3 ± 2.2 | 10.2 ± 2.3 | 10.5 ± 2.4 | 10.0 ± 2.3 | 9.9 ± 2.2 |
| B180 (%BW) | 10.1 ± 2.3 | 10.2 ± 2.3 | 10.3 ± 2.2 | 10.0 ± 2.3 | 9.8 ± 2.2 |
| B225 (%BW) | 10.6 ± 2.7 | 10.6 ± 2.9 | 10.8 ± 2.5 | 10.4 ± 2.7 | 10.2 ± 2.5 |
| TP (%BW) | 12.1 ± 3.0 | 12.2 ± 3.4 | 12.0 ± 2.9 | 11.8 ± 3.3 | 11.6 ± 2.7 |
| TC (%BW) | 11.9 ± 2.7 | 12.0 ± 3.0 | 11.8 ± 3.1 | 11.4 ± 3.2 | 11.1 ± 2.4 |
| D225 (%BW) | 8.9 ± 3.4 | 8.9 ± 3.7 | 9.2 ± 2.7 | 8.9 ± 3.4 | 8.5 ± 3.0 |
| D180 (%BW) | 8.8 ± 3.9 | 9.2 ± 4.2 | 9.1 ± 3.4 | 9.4 ± 3.9 | 8.9 ± 3.8 |
| D135 (%BW) | 7.8 ± 4.0 | 8.2 ± 4.1 | 8.1 ± 3.7 | 8.7 ± 3.8 | 8.7 ± 4.0 |
| D90 (%BW) | 5.3 ± 3.9 | 5.8 ± 4.1 | 5.7 ± 3.8 | 6.5 ± 4.1 | 7.1 ± 4.5 |
| D45 (%BW) | 2.6 ± 4.0 | 3.2 ± 4.5 | 3.3 ± 4.2 | 3.9 ± 4.8 | 4.7 ± 5.2 |
| I (%BW) | 1.0 ± 4.2 | 1.8 ± 4.9 | 2.1 ± 4.6 | 2.5 ± 5.2 | 3.5 ± 5.6 |

**Table S5.** Left foot anteroposterior ground reaction force (*GRF_X,L_*) (mean ± SD) for 5 mediolateral ball positions over fourteen swing events analyzed: Address (A), backswing 45° (B45), backswing 90° (B90), backswing 135° (B135), backswing 180° (B180), backswing 225° (B225), transition of the pelvis (TP), transition of the club (TC), downswing 225° (D225), downswing 180° (D180), downswing 135° (D135), downswing 90° (D90), downswing 45° (D45), and impact (I). LF, LH, R0, RH, and RF stand for left-full position (4.27 cm left from the reference), left-half position (2.14 cm left from the reference), reference ball position (i.e. preferred ball position), right-half position (2.14 cm right from the reference), and right-full position (4.27 cm right from the reference), respectively. %BW: percentages of body weight.

|  | **LF** | **LH** | **R0** | **RH** | **RF** |
| --- | --- | --- | --- | --- | --- |
| A (%BW) | 1.3 ± 1.2 | 1.5 ± 1.4 | 1.8 ± 1.4 | 1.6 ± 1.6 | 1.7 ± 1.3 |
| B45 (%BW) | 6.5 ± 1.7 | 6.5 ± 1.8 | 6.6 ± 1.7 | 6.5 ± 1.8 | 6.5 ± 1.9 |
| B90 (%BW) | 3.7 ± 1.1 | 3.8 ± 1.1 | 3.8 ± 1.1 | 3.7 ± 1.1 | 3.8 ± 1.1 |
| B135 (%BW) | 1.5 ± 1.4 | 1.6 ± 1.5 | 1.6 ± 1.2 | 1.5 ± 1.4 | 1.7 ± 1.3 |
| B180 (%BW) | 0.8 ± 1.5 | 0.8 ± 1.4 | 0.7 ± 1.3 | 0.8 ± 1.4 | 1.0 ± 1.3 |
| B225 (%BW) | 0.4 ± 1.6 | 0.5 ± 1.6 | 0.5 ± 1.3 | 0.5 ± 1.5 | 0.7 ± 1.6 |
| TP (%BW) | –1.3 ± 1.9 | –1.1 ± 2.1 | –1.3 ± 1.6 | –1.1 ± 2.2 | –1.0 ± 2.0 |
| TC (%BW) | –6.1 ± 4.0 | –5.8 ± 4.3 | –5.9 ± 3.9 | –5.7 ± 4.2 | –5.9 ± 4.1 |
| D225 (%BW) | –22.4 ± 5.1 | –22.5 ± 4.9 | –22.0 ± 4.6 | –21.6 ± 4.6 | –22.0 ± 4.7 |
| D180 (%BW) | –16.6 ± 4.6 | –17.5 ± 5.5 | –17.9 ± 5.3 | –17.5 ± 5.7 | –17.0 ± 5.3 |
| D135 (%BW) | –8.9 ± 3.9 | –9.4 ± 3.8 | –9.5 ± 4.1 | –9.5 ± 4.1 | –8.8 ± 4.1 |
| D90 (%BW) | –4.8 ± 4.1 | –5.0 ± 4.5 | –4.7 ± 4.5 | –4.9 ± 4.4 | –4.4 ± 4.5 |
| D45 (%BW) | 0.0 ± 3.9 | –0.2 ± 4.4 | –0.1 ± 4.1 | –0.3 ± 4.1 | 0.2 ± 4.1 |
| I (%BW) | 2.7 ± 3.6 | 2.7 ± 3.7 | 2.6 ± 3.6 | 2.4 ± 3.5 | 2.7 ± 3.6 |

**Table S6.** Right foot anteroposterior ground reaction force (*GRF_X,R_*) (mean ± SD) for 5 mediolateral ball positions over fourteen swing events analyzed: Address (A), backswing 45° (B45), backswing 90° (B90), backswing 135° (B135), backswing 180° (B180), backswing 225° (B225), transition of the pelvis (TP), transition of the club (TC), downswing 225° (D225), downswing 180° (D180), downswing 135° (D135), downswing 90° (D90), downswing 45° (D45), and impact (I). LF, LH, R0, RH, and RF stand for left-full position (4.27 cm left from the reference), left-half position (2.14 cm left from the reference), reference ball position (i.e. preferred ball position), right-half position (2.14 cm right from the reference), and right-full position (4.27 cm right from the reference), respectively. %BW: percentages of body weight.

|  | **LF** | **LH** | **R0** | **RH** | **RF** |
| --- | --- | --- | --- | --- | --- |
| A (%BW) | –1.5 ± 1.0 | –1.6 ± 1.2 | –2.0 ± 1.2 | –1.8 ± 1.5 | –1.9 ± 1.2 |
| B45 (%BW) | –5.9 ± 1.6 | –5.9 ± 1.5 | –6.0 ± 1.5 | –5.9 ± 1.6 | –6.0 ± 1.7 |
| B90 (%BW) | –3.3 ± 1.2 | –3.5 ± 1.1 | –3.2 ± 1.2 | –3.4 ± 1.2 | –3.5 ± 1.2 |
| B135 (%BW) | –1.7 ± 1.1 | –1.7 ± 1.1 | –1.6 ± 1.1 | –1.6 ± 1.2 | –1.9 ± 1.0 |
| B180 (%BW) | –1.1 ± 1.1 | –1.1 ± 1.3 | –1.0 ± 1.1 | –1.2 ± 1.2 | –1.3 ± 1.1 |
| B225 (%BW) | –0.7 ± 1.2 | –0.6 ± 1.4 | –0.7 ± 1.2 | –0.8 ± 1.4 | –1.0 ± 1.2 |
| TP (%BW) | 0.9 ± 1.3 | 0.7 ± 1.6 | 1.0 ± 1.3 | 0.7 ± 1.6 | 0.8 ± 1.5 |
| TC (%BW) | 5.4 ± 3.5 | 5.0 ± 4.0 | 5.2 ± 3.4 | 5.1 ± 4.0 | 5.5 ± 3.8 |
| D225 (%BW) | 16.8 ± 2.4 | 17.0 ± 2.2 | 17.0 ± 2.2 | 17.1 ± 2.3 | 17.2 ± 2.3 |
| D180 (%BW) | 15.4 ± 3.1 | 15.6 ± 3.1 | 16.1 ± 2.8 | 15.8 ± 3.1 | 15.9 ± 3.5 |
| D135 (%BW) | 11.6 ± 3.2 | 11.6 ± 3.3 | 12.6 ± 3.3 | 11.9 ± 3.5 | 12.0 ± 3.8 |
| D90 (%BW) | 7.3 ± 3.5 | 7.0 ± 3.3 | 8.1 ± 3.4 | 7.2 ± 3.2 | 7.1 ± 4.0 |
| D45 (%BW) | 4.1 ± 3.4 | 3.9 ± 3.4 | 4.7 ± 3.5 | 4.0 ± 3.3 | 3.5 ± 4.0 |
| I (%BW) | 2.4 ± 3.0 | 2.2 ± 3.0 | 2.9 ± 3.2 | 2.2 ± 3.1 | 1.8 ± 3.7 |

**Table S7.** Mediolateral centre of pressure position (*COP_Y_*) (mean ± SD) for 5 mediolateral ball positions over fourteen swing events analyzed: Address (A), backswing 45° (B45), backswing 90° (B90), backswing 135° (B135), backswing 180° (B180), backswing 225° (B225), transition of the pelvis (TP), transition of the club (TC), downswing 225° (D225), downswing 180° (D180), downswing 135° (D135), downswing 90° (D90), downswing 45° (D45), and impact (I). LF, LH, R0, RH, and RF stand for left-full position (4.27 cm left from the reference), left-half position (2.14 cm left from the reference), reference ball position (i.e. preferred ball position), right-half position (2.14 cm right from the reference), and right-full position (4.27 cm right from the reference), respectively.

|  | **LF** | **LH** | **R0** | **RH** | **RF** |
| --- | --- | --- | --- | --- | --- |
| Address (mm) | 592.7 ± 29.6 | 587.9 ± 34.6 | 582.4 ± 33.3 | 575.9 ± 30.7 | 567.8 ± 28.9 |
| B45 (mm) | 502.0 ± 29.7 | 498.4 ± 30.6 | 491.9 ± 30.9 | 488.1 ± 30.5 | 488.3 ± 27.6 |
| B90 (mm) | 465.0 ± 36.3 | 461.7 ± 37.4 | 458.5 ± 34.4 | 453.5 ± 35.4 | 453.1 ± 32.9 |
| B135 (mm) | 457.7 ± 42.2 | 456.1 ± 42.8 | 453.7 ± 38.8 | 450.3 ± 40.3 | 449.8 ± 39.7 |
| B180 (mm) | 462.0 ± 41.8 | 460.2 ± 42.6 | 459.7 ± 39.0 | 456.0 ± 42.5 | 456.2 ± 41.2 |
| B225 (mm) | 468.5 ± 44.8 | 466.0 ± 45.5 | 467.0 ± 42.8 | 464.8 ± 47.8 | 464.9 ± 44.4 |
| TP (mm) | 462.2 ± 52.3 | 459.7 ± 52.5 | 464.0 ± 50.5 | 459.8 ± 55.0 | 460.0 ± 49.6 |
| TC (mm) | 471.4 ± 41.3 | 470.2 ± 41.5 | 473.9 ± 40.8 | 469.7 ± 44.5 | 468.5 ± 37.7 |
| D225 (mm) | 604.4 ± 40.0 | 600.9 ± 38.1 | 591.3 ± 31.6 | 591.5 ± 39.9 | 592.0 ± 39.8 |
| D180 (mm) | 639.0 ± 35.8 | 631.6 ± 39.7 | 627.0 ± 34.0 | 619.9 ± 39.3 | 616.6 ± 40.0 |
| D135 (mm) | 664.9 ± 42.7 | 655.2 ± 48.5 | 652.8 ± 42.0 | 640.7 ± 46.4 | 633.7 ± 45.6 |
| D90 (mm) | 681.6 ± 49.2 | 671.8 ± 53.9 | 669.4 ± 48.2 | 656.5 ± 52.7 | 643.5 ± 51.9 |
| D45 (mm) | 692.5 ± 51.6 | 683.5 ± 54.1 | 680.4 ± 49.0 | 668.0 ± 53.9 | 651.4 ± 54.0 |
| Impact (mm) | 695.9 ± 53.7 | 687.4 ± 54.6 | 683.9 ± 48.8 | 671.4 ± 53.6 | 653.2 ± 55.4 |

**Table S8.** Anteroposterior centre of pressure position (*COP_X_*) (mean ± SD) for 5 mediolateral ball positions over fourteen swing events analyzed: Address (A), backswing 45° (B45), backswing 90° (B90), backswing 135° (B135), backswing 180° (B180), backswing 225° (B225), transition of the pelvis (TP), transition of the club (TC), downswing 225° (D225), downswing 180° (D180), downswing 135° (D135), downswing 90° (D90), downswing 45° (D45), and impact (I). LF, LH, R0, RH, and RF stand for left-full position (4.27 cm left from the reference), left-half position (2.14 cm left from the reference), reference ball position (i.e. preferred ball position), right-half position (2.14 cm right from the reference), and right-full position (4.27 cm right from the reference), respectively.

|  | **LF** | **LH** | **R0** | **RH** | **RF** |
| --- | --- | --- | --- | --- | --- |
| A (mm) | 245.4 ± 29.5 | 246.1 ± 32.3 | 248.0 ± 30.6 | 248.4 ± 30.3 | 246.6 ± 30.9 |
| B45 (mm) | 244.4 ± 33.2 | 245.0 ± 34.3 | 246.5 ± 35.9 | 248.0 ± 33.5 | 246.0 ± 33.3 |
| B90 (mm) | 245.4 ± 35.1 | 245.1 ± 35.1 | 245.6 ± 36.2 | 248.5 ± 33.7 | 246.6 ± 33.5 |
| B135 (mm) | 242.6 ± 35.7 | 241.9 ± 36.9 | 242.6 ± 35.6 | 245.0 ± 34.2 | 243.7 ± 34.3 |
| B180 (mm) | 239.6 ± 35.8 | 238.5 ± 36.2 | 240.3 ± 33.9 | 242.5 ± 33.4 | 240.3 ± 34.1 |
| B225 (mm) | 237.7 ± 36.7 | 236.7 ± 35.8 | 237.9 ± 34.4 | 240.5 ± 34.7 | 238.7 ± 36.0 |
| TP (mm) | 239.7 ± 37.1 | 239.6 ± 37.0 | 241.2 ± 36.2 | 242.0 ± 37.2 | 239.4 ± 37.5 |
| TC (mm) | 244.0 ± 37.5 | 244.3 ± 36.0 | 246.3 ± 36.4 | 245.9 ± 36.8 | 242.7 ± 37.5 |
| D225 (mm) | 266.9 ± 31.9 | 265.8 ± 30.7 | 265.7 ± 32.8 | 264.2 ± 33.7 | 262.6 ± 33.0 |
| D180 (mm) | 261.9 ± 35.8 | 262.7 ± 35.5 | 262.4 ± 37.3 | 263.5 ± 37.4 | 261.4 ± 37.5 |
| D135 (mm) | 253.4 ± 39.6 | 255.4 ± 41.0 | 253.5 ± 42.1 | 257.7 ± 42.0 | 255.3 ± 42.0 |
| D90 (mm) | 248.4 ± 40.2 | 250.2 ± 42.3 | 248.5 ± 43.9 | 253.4 ± 43.2 | 252.7 ± 43.0 |
| D45 (mm) | 242.0 ± 39.2 | 243.3 ± 41.6 | 243.2 ± 43.8 | 247.7 ± 42.1 | 248.1 ± 41.3 |
| I (mm) | 239.7 ± 38.3 | 240.9 ± 41.3 | 241.4 ± 43.3 | 245.7 ± 40.7 | 246.6 ± 40.6 |

**Table S9.** Shoulder angle (*A_S_*) (mean ± SD) for 5 mediolateral ball positions over fourteen swing events analyzed: Address (A), backswing 45° (B45), backswing 90° (B90), backswing 135° (B135), backswing 180° (B180), backswing 225° (B225), transition of the pelvis (TP), transition of the club (TC), downswing 225° (D225), downswing 180° (D180), downswing 135° (D135), downswing 90° (D90), downswing 45° (D45), and impact (I). LF, LH, R0, RH, and RF stand for left-full position (4.27 cm left from the reference), left-half position (2.14 cm left from the reference), reference ball position (i.e. preferred ball position), right-half position (2.14 cm right from the reference), and right-full position (4.27 cm right from the reference), respectively.

|  | **LF** | **LH** | **R0** | **RH** | **RF** |
| --- | --- | --- | --- | --- | --- |
| A (°) | 0.7 ± 2.4 | 0.1 ± 2.7 | 0.2 ± 3.0 | –0.4 ± 2.8 | –0.8 ± 2.8 |
| B45 (°) | –17.3 ± 5.0 | –17.6 ± 4.9 | –17.7 ± 5.2 | –17.8 ± 5.1 | –17.9 ± 5.4 |
| B90 (°) | –40.3 ± 6.9 | –40.7 ± 7.0 | –40.8 ± 7.0 | –41.0 ± 7.4 | –41.3 ± 7.3 |
| B135 (°) | –62.1 ± 8.5 | –62.5 ± 8.6 | –62.1 ± 8.1 | –62.6 ± 8.9 | –63.0 ± 8.8 |
| B180 (°) | –77.3 ± 9.5 | –77.7 ± 9.4 | –77.4 ± 8.7 | –77.9 ± 9.6 | –78.3 ± 9.4 |
| B225 (°) | –91.6 ± 8.8 | –92.0 ± 8.7 | –91.7 ± 8.1 | –92.3 ± 8.8 | –92.7 ± 8.5 |
| TP (°) | –102.8 ± 7.6 | –102.9 ± 7.8 | –102.5 ± 7.0 | –103.6 ± 8.0 | –104.3 ± 7.6 |
| TC (°) | –102.8 ± 7.9 | –102.8 ± 8.0 | –102.5 ± 7.4 | –103.6 ± 8.1 | –104.3 ± 7.8 |
| D225 (°) | –58.3 ± 12.2 | –59.0 ± 12.6 | –60.3 ± 11.5 | –59.9 ± 12.0 | –59.5 ± 12.0 |
| D180 (°) | –36.7 ± 8.1 | –37.3 ± 8.4 | –38.1 ± 7.7 | –38.2 ± 8.3 | –38.2 ± 8.2 |
| D135 (°) | –21.7 ± 6.6 | –22.5 ± 6.8 | –23.2 ± 6.3 | –23.4 ± 6.7 | –23.5 ± 6.9 |
| D90 (°) | –8.9 ± 6.3 | –9.5 ± 6.5 | –10.2 ± 6.0 | –10.5 ± 6.3 | –10.8 ± 6.6 |
| D45 (°) | 2.4 ± 6.6 | 1.6 ± 6.6 | 1.7 ± 6.2 | 0.7 ± 6.8 | 0.4 ± 7.0 |
| I (°) | 8.9 ± 6.9 | 8.1 ± 6.8 | 7.3 ± 6.6 | 6.9 ± 6.8 | 6.4 ± 7.2 |

**Table S10.** Pelvis angle (*A_P_*) (mean ± SD) for 5 mediolateral ball positions over fourteen swing events analyzed: Address (A), backswing 45° (B45), backswing 90° (B90), backswing 135° (B135), backswing 180° (B180), backswing 225° (B225), transition of the pelvis (TP), transition of the club (TC), downswing 225° (D225), downswing 180° (D180), downswing 135° (D135), downswing 90° (D90), downswing 45° (D45), and impact (I). LF, LH, R0, RH, and RF stand for left-full position (4.27 cm left from the reference), left-half position (2.14 cm left from the reference), reference ball position (i.e. preferred ball position), right-half position (2.14 cm right from the reference), and right-full position (4.27 cm right from the reference), respectively.

|  | **LF** | **LH** | **R0** | **RH** | **RF** |
| --- | --- | --- | --- | --- | --- |
| A (°) | 1.2 ± 3.2 | 0.8 ± 3.4 | 0.7 ± 3.4 | 1.0 ± 3.1 | 0.5 ± 3.2 |
| B45 (°) | –14.3 ± 3.4 | –14.9 ± 3.6 | –15.0 ± 4.2 | –14.8 ± 3.7 | –14.6 ± 3.3 |
| B90 (°) | –26.2 ± 4.7 | –26.8 ± 4.6 | –26.8 ± 5.1 | –26.8 ± 4.9 | –26.7 ± 4.6 |
| B135 (°) | –35.4 ± 5.7 | –35.8 ± 5.5 | –35.7 ± 5.7 | –35.8 ± 5.8 | –36.0 ± 5.5 |
| B180 (°) | –41.6 ± 6.2 | –42.0 ± 6.0 | –41.8 ± 6.1 | –42.0 ± 6.2 | –42.3 ± 5.9 |
| B225 (°) | –47.6 ± 6.6 | –47.9 ± 6.5 | –47.5 ± 6.5 | –47.9 ± 6.6 | –48.4 ± 6.3 |
| TP (°) | –51.9 ± 6.7 | –52.0 ± 6.6 | –51.5 ± 6.4 | –52.2 ± 6.6 | –52.9 ± 6.3 |
| TC (°) | –48.7 ± 7.1 | –48.8 ± 6.7 | –48.5 ± 6.7 | –49.0 ± 6.3 | –49.8 ± 6.3 |
| D225 (°) | –8.4 ± 10.9 | –9.2 ± 10.7 | –10.2 ± 10.8 | –9.6 ± 10.2 | –9.5 ± 10.4 |
| D180 (°) | 8.0 ± 9.2 | 7.3 ± 8.9 | 6.5 ± 9.0 | 6.7 ± 8.5 | 6.6 ± 8.9 |
| D135 (°) | 19.6 ± 9.3 | 18.7 ± 8.9 | 18.0 ± 9.0 | 18.1 ± 8.6 | 17.9 ± 9.1 |
| D90 (°) | 29.0 ± 9.7 | 28.3 ± 9.4 | 27.4 ± 9.6 | 27.5 ± 9.0 | 27.2 ± 9.5 |
| D45 (°) | 36.2 ± 10.1 | 35.4 ± 9.7 | 34.7 ± 9.9 | 34.7 ± 9.4 | 34.5 ± 9.9 |
| I (°) | 39.6 ± 10.2 | 39.0 ± 10.0 | 38.0 ± 10.0 | 38.1 ± 9.5 | 37.8 ± 10.0 |

**Table S11.** Trunk angle (*A_T_*) (mean ± SD) for 5 mediolateral ball positions over fourteen swing events analyzed: Address (A), backswing 45° (B45), backswing 90° (B90), backswing 135° (B135), backswing 180° (B180), backswing 225° (B225), transition of the pelvis (TP), transition of the club (TC), downswing 225° (D225), downswing 180° (D180), downswing 135° (D135), downswing 90° (D90), downswing 45° (D45), and impact (I). LF, LH, R0, RH, and RF stand for left-full position (4.27 cm left from the reference), left-half position (2.14 cm left from the reference), reference ball position (i.e. preferred ball position), right-half position (2.14 cm right from the reference), and right-full position (4.27 cm right from the reference), respectively.

|  | **LF** | **LH** | **R0** | **RH** | **RF** |
| --- | --- | --- | --- | --- | --- |
| A (°) | 40.7 ± 2.9 | 40.6 ± 2.7 | 40.6 ± 2.7 | 40.5 ± 2.7 | 40.4 ± 2.8 |
| B45 (°) | 39.7 ± 3.1 | 39.6 ± 2.9 | 39.7 ± 3.0 | 39.6 ± 3.0 | 39.6 ± 3.0 |
| B90 (°) | 39.4 ± 3.1 | 39.3 ± 2.9 | 39.4 ± 3.0 | 39.4 ± 3.0 | 39.4 ± 3.0 |
| B135 (°) | 39.3 ± 3.2 | 39.3 ± 3.0 | 39.4 ± 3.1 | 39.3 ± 3.1 | 39.3 ± 3.2 |
| B180 (°) | 39.2 ± 3.4 | 39.1 ± 3.2 | 39.3 ± 3.2 | 39.2 ± 3.2 | 39.2 ± 3.3 |
| B225 (°) | 39.0 ± 3.4 | 39.0 ± 3.3 | 39.1 ± 3.3 | 39.0 ± 3.3 | 39.0 ± 3.4 |
| TP (°) | 39.0 ± 3.6 | 38.9 ± 3.6 | 39.1 ± 3.5 | 39.0 ± 3.5 | 38.9 ± 3.6 |
| TC (°) | 39.9 ± 3.8 | 39.9 ± 3.7 | 39.9 ± 3.6 | 40.0 ± 3.6 | 39.9 ± 3.7 |
| D225 (°) | 41.9 ± 3.4 | 41.9 ± 3.4 | 42.0 ± 3.4 | 41.9 ± 3.3 | 41.9 ± 3.5 |
| D180 (°) | 40.0 ± 3.5 | 40.2 ± 3.5 | 40.3 ± 3.6 | 40.2 ± 3.4 | 40.1 ± 3.6 |
| D135 (°) | 38.1 ± 3.7 | 38.3 ± 3.8 | 38.5 ± 3.8 | 38.3 ± 3.6 | 38.1 ± 3.9 |
| D90 (°) | 36.4 ± 4.0 | 36.6 ± 4.0 | 36.8 ± 4.0 | 36.5 ± 3.8 | 36.4 ± 4.2 |
| D45 (°) | 35.0 ± 4.3 | 35.2 ± 4.2 | 35.4 ± 4.2 | 35.1 ± 4.1 | 34.9 ± 4.5 |
| I (°) | 34.4 ± 4.5 | 34.5 ± 4.4 | 34.7 ± 4.3 | 34.4 ± 4.2 | 34.2 ± 4.6 |

**Table S12.** Left knee angle (*A_K,L_*) (mean ± SD) for 5 mediolateral ball positions over fourteen swing events analyzed: Address (A), backswing 45° (B45), backswing 90° (B90), backswing 135° (B135), backswing 180° (B180), backswing 225° (B225), transition of the pelvis (TP), transition of the club (TC), downswing 225° (D225), downswing 180° (D180), downswing 135° (D135), downswing 90° (D90), downswing 45° (D45), and impact (I). LF, LH, R0, RH, and RF stand for left-full position (4.27 cm left from the reference), left-half position (2.14 cm left from the reference), reference ball position (i.e. preferred ball position), right-half position (2.14 cm right from the reference), and right-full position (4.27 cm right from the reference), respectively.

|  | **LF** | **LH** | **R0** | **RH** | **RF** |
| --- | --- | --- | --- | --- | --- |
| A (°) | 155.9 ± 7.9 | 156.2 ± 8.1 | 156.5 ± 7.8 | 156.7 ± 8.0 | 156.3 ± 8.1 |
| B45 (°) | 154.5 ± 8.4 | 154.5 ± 8.5 | 154.8 ± 8.3 | 155.1 ± 8.4 | 154.8 ± 8.7 |
| B90 (°) | 150.8 ± 8.8 | 151.0 ± 8.8 | 151.4 ± 8.8 | 151.6 ± 8.7 | 151.4 ± 9.2 |
| B135 (°) | 148.5 ± 9.3 | 148.8 ± 9.1 | 149.2 ± 9.2 | 149.4 ± 9.1 | 149.1 ± 9.5 |
| B180 (°) | 147.5 ± 9.5 | 148.0 ± 9.3 | 148.2 ± 9.5 | 148.5 ± 9.4 | 148.3 ± 9.7 |
| B225 (°) | 147.1 ± 9.7 | 147.6 ± 9.6 | 147.7 ± 9.6 | 148.1 ± 9.5 | 147.8 ± 9.8 |
| TP (°) | 145.3 ± 9.7 | 145.8 ± 9.7 | 146.0 ± 9.6 | 146.6 ± 9.7 | 146.2 ± 10.0 |
| TC (°) | 142.6 ± 9.0 | 143.0 ± 9.0 | 143.3 ± 8.7 | 143.9 ± 8.9 | 143.5 ± 9.2 |
| D225 (°) | 136.0 ± 8.1 | 136.0 ± 7.9 | 136.2 ± 7.8 | 136.7 ± 8.0 | 136.5 ± 8.0 |
| D180 (°) | 140.0 ± 8.2 | 139.9 ± 8.0 | 139.9 ± 7.9 | 140.3 ± 8.0 | 140.4 ± 8.1 |
| D135 (°) | 145.2 ± 7.9 | 145.0 ± 7.6 | 145.0 ± 7.8 | 145.3 ± 7.7 | 145.5 ± 7.9 |
| D90 (°) | 150.6 ± 7.3 | 150.7 ± 7.1 | 150.5 ± 7.4 | 150.8 ± 7.1 | 150.9 ± 7.3 |
| D45 (°) | 155.3 ± 7.0 | 155.4 ± 6.7 | 155.3 ± 7.2 | 155.7 ± 6.7 | 155.9 ± 6.9 |
| I (°) | 157.9 ± 6.7 | 158.1 ± 6.5 | 157.6 ± 7.2 | 158.2 ± 6.4 | 158.3 ± 6.8 |

**Table S13.** Right knee angle (*A_K,R_*) (mean ± SD) for 5 mediolateral ball positions over fourteen swing events analyzed: Address (A), backswing 45° (B45), backswing 90° (B90), backswing 135° (B135), backswing 180° (B180), backswing 225° (B225), transition of the pelvis (TP), transition of the club (TC), downswing 225° (D225), downswing 180° (D180), downswing 135° (D135), downswing 90° (D90), downswing 45° (D45), and impact (I). LF, LH, R0, RH, and RF stand for left-full position (4.27 cm left from the reference), left-half position (2.14 cm left from the reference), reference ball position (i.e. preferred ball position), right-half position (2.14 cm right from the reference), and right-full position (4.27 cm right from the reference), respectively.

|  | **LF** | **LH** | **R0** | **RH** | **RF** |
| --- | --- | --- | --- | --- | --- |
| A (°) | 153.1 ± 7.1 | 153.4 ± 7.4 | 152.8 ± 7.4 | 152.9 ± 7.7 | 152.7 ± 7.4 |
| B45 (°) | 152.9 ± 7.7 | 153.1 ± 7.8 | 152.8 ± 7.8 | 152.6 ± 7.8 | 152.5 ± 7.8 |
| B90 (°) | 154.4 ± 7.8 | 154.2 ± 8.0 | 154.1 ± 8.0 | 153.9 ± 7.9 | 153.9 ± 7.9 |
| B135 (°) | 154.6 ± 7.8 | 154.4 ± 8.0 | 154.2 ± 7.9 | 154.1 ± 8.0 | 154.0 ± 7.8 |
| B180 (°) | 154.4 ± 7.8 | 154.2 ± 7.9 | 153.9 ± 7.9 | 153.9 ± 8.0 | 153.7 ± 7.9 |
| B225 (°) | 154.3 ± 7.7 | 154.1 ± 7.8 | 153.6 ± 7.8 | 153.7 ± 8.0 | 153.5 ± 7.8 |
| TP (°) | 154.5 ± 7.7 | 154.2 ± 7.9 | 153.9 ± 7.8 | 153.8 ± 8.1 | 153.7 ± 7.8 |
| TC (°) | 154.0 ± 7.8 | 153.7 ± 8.2 | 153.4 ± 8.0 | 153.3 ± 8.3 | 153.1 ± 7.9 |
| D225 (°) | 147.8 ± 10.3 | 147.5 ± 10.5 | 147.7 ± 10.1 | 147.4 ± 10.6 | 146.8 ± 10.1 |
| D180 (°) | 147.3 ± 11.0 | 147.1 ± 10.9 | 147.2 ± 10.7 | 146.9 ± 11.0 | 146.5 ± 10.6 |
| D135 (°) | 148.2 ± 11.0 | 148.0 ± 10.9 | 148.0 ± 10.6 | 147.7 ± 10.9 | 147.4 ± 10.6 |
| D90 (°) | 149.7 ± 10.5 | 149.6 ± 10.3 | 149.5 ± 10.0 | 149.3 ± 10.2 | 148.9 ± 10.0 |
| D45 (°) | 151.3 ± 10.0 | 151.2 ± 9.6 | 151.2 ± 9.1 | 151.0 ± 9.5 | 150.7 ± 9.3 |
| I (°) | 152.4 ± 9.8 | 152.2 ± 9.3 | 152.2 ± 8.7 | 151.9 ± 9.2 | 151.6 ± 9.0 |

**Table S14.** Left ankle angle (*A_A,L_*) (mean ± SD) for 5 mediolateral ball positions over fourteen swing events analyzed: Address (A), backswing 45° (B45), backswing 90° (B90), backswing 135° (B135), backswing 180° (B180), backswing 225° (B225), transition of the pelvis (TP), transition of the club (TC), downswing 225° (D225), downswing 180° (D180), downswing 135° (D135), downswing 90° (D90), downswing 45° (D45), and impact (I). LF, LH, R0, RH, and RF stand for left-full position (4.27 cm left from the reference), left-half position (2.14 cm left from the reference), reference ball position (i.e. preferred ball position), right-half position (2.14 cm right from the reference), and right-full position (4.27 cm right from the reference), respectively.

|  | **LF** | **LH** | **R0** | **RH** | **RF** |
| --- | --- | --- | --- | --- | --- |
| A (°) | 83.7 ± 4.4 | 83.8 ± 4.2 | 83.7 ± 4.2 | 83.8 ± 4.2 | 83.6 ± 4.5 |
| B45 (°) | 80.1 ± 4.4 | 80.0 ± 4.2 | 79.8 ± 4.2 | 80.1 ± 4.1 | 80.1 ± 4.5 |
| B90 (°) | 75.5 ± 4.7 | 75.6 ± 4.4 | 75.4 ± 4.7 | 75.7 ± 4.5 | 75.7 ± 4.9 |
| B135 (°) | 72.2 ± 5.2 | 72.3 ± 4.7 | 72.2 ± 5.0 | 72.5 ± 4.8 | 72.4 ± 5.3 |
| B180 (°) | 70.4 ± 5.4 | 70.5 ± 5.0 | 70.3 ± 5.3 | 70.6 ± 5.1 | 70.6 ± 5.5 |
| B225 (°) | 69.1 ± 5.5 | 69.2 ± 5.2 | 69.0 ± 5.4 | 69.3 ± 5.2 | 69.3 ± 5.6 |
| TP (°) | 68.0 ± 5.4 | 68.2 ± 5.1 | 68.0 ± 5.3 | 68.4 ± 5.0 | 68.3 ± 5.5 |
| TC (°) | 67.7 ± 5.3 | 67.8 ± 5.1 | 67.8 ± 5.2 | 68.1 ± 4.9 | 68.0 ± 5.4 |
| D225 (°) | 73.2 ± 6.6 | 73.2 ± 6.3 | 72.5 ± 6.3 | 73.1 ± 6.0 | 73.1 ± 6.5 |
| D180 (°) | 78.0 ± 6.3 | 78.0 ± 6.0 | 77.3 ± 6.0 | 77.8 ± 5.7 | 77.9 ± 6.3 |
| D135 (°) | 82.0 ± 5.8 | 82.0 ± 5.6 | 81.4 ± 5.6 | 81.7 ± 5.3 | 81.9 ± 6.0 |
| D90 (°) | 85.7 ± 5.6 | 85.8 ± 5.4 | 85.3 ± 5.4 | 85.5 ± 5.1 | 85.5 ± 5.8 |
| D45 (°) | 89.1 ± 5.7 | 89.2 ± 5.6 | 88.6 ± 5.7 | 88.8 ± 5.2 | 89.0 ± 6.1 |
| I (°) | 91.0 ± 6.1 | 91.1 ± 5.9 | 90.3 ± 6.2 | 90.6 ± 5.5 | 90.8 ± 6.4 |

**Table S15.** Right ankle angle (*A_A,R_*) (mean ± SD) for 5 mediolateral ball positions over fourteen swing events analyzed: Address (A), backswing 45° (B45), backswing 90° (B90), backswing 135° (B135), backswing 180° (B180), backswing 225° (B225), transition of the pelvis (TP), transition of the club (TC), downswing 225° (D225), downswing 180° (D180), downswing 135° (D135), downswing 90° (D90), downswing 45° (D45), and impact (I). LF, LH, R0, RH, and RF stand for left-full position (4.27 cm left from the reference), left-half position (2.14 cm left from the reference), reference ball position (i.e. preferred ball position), right-half position (2.14 cm right from the reference), and right-full position (4.27 cm right from the reference), respectively.

|  | **LF** | **LH** | **R0** | **RH** | **RF** |
| --- | --- | --- | --- | --- | --- |
| A (°) | 83.1 ± 3.7 | 83.3 ± 3.6 | 83.2 ± 3.6 | 83.1 ± 3.9 | 83.1 ± 3.7 |
| B45 (°) | 85.8 ± 4.4 | 86.0 ± 4.2 | 86.0 ± 4.4 | 85.8 ± 4.4 | 85.6 ± 4.4 |
| B90 (°) | 88.0 ± 4.7 | 88.1 ± 4.4 | 88.1 ± 4.5 | 87.9 ± 4.5 | 87.8 ± 4.8 |
| B135 (°) | 88.8 ± 4.8 | 88.8 ± 4.4 | 88.8 ± 4.4 | 88.7 ± 4.5 | 88.6 ± 4.8 |
| B180 (°) | 89.1 ± 4.8 | 89.1 ± 4.5 | 89.0 ± 4.5 | 89.0 ± 4.5 | 89.0 ± 4.9 |
| B225 (°) | 89.6 ± 4.7 | 89.6 ± 4.6 | 89.3 ± 4.5 | 89.4 ± 4.6 | 89.4 ± 5.0 |
| TP (°) | 90.4 ± 4.8 | 90.3 ± 4.8 | 90.0 ± 4.5 | 90.2 ± 4.7 | 90.2 ± 5.0 |
| TC (°) | 89.8 ± 4.7 | 89.8 ± 4.8 | 89.5 ± 4.4 | 89.6 ± 4.6 | 89.5 ± 4.8 |
| D225 (°) | 82.1 ± 6.0 | 82.2 ± 6.1 | 82.2 ± 5.5 | 82.0 ± 6.0 | 81.4 ± 5.8 |
| D180 (°) | 81.1 ± 7.3 | 81.0 ± 7.4 | 80.7 ± 6.7 | 80.6 ± 7.2 | 80.0 ± 7.0 |
| D135 (°) | 81.6 ± 8.1 | 81.3 ± 8.1 | 80.7 ± 7.3 | 80.7 ± 8.0 | 80.1 ± 7.7 |
| D90 (°) | 83.4 ± 8.0 | 82.9 ± 7.9 | 82.0 ± 7.2 | 82.1 ± 7.9 | 81.6 ± 7.7 |
| D45 (°) | 85.8 ± 7.6 | 85.1 ± 7.3 | 84.0 ± 6.5 | 84.3 ± 7.2 | 83.9 ± 7.2 |
| I (°) | 87.4 ± 7.2 | 86.7 ± 6.8 | 85.3 ± 6.1 | 85.8 ± 6.7 | 85.4 ± 7.0 |

**Table S16.** Mediolateral centre of mass position (*COM_Y_*) (mean ± SD) for 5 mediolateral ball positions over fourteen swing events analyzed: Address (A), backswing 45° (B45), backswing 90° (B90), backswing 135° (B135), backswing 180° (B180), backswing 225° (B225), transition of the pelvis (TP), transition of the club (TC), downswing 225° (D225), downswing 180° (D180), downswing 135° (D135), downswing 90° (D90), downswing 45° (D45), and impact (I). LF, LH, R0, RH, and RF stand for left-full position (4.27 cm left from the reference), left-half position (2.14 cm left from the reference), reference ball position (i.e. preferred ball position), right-half position (2.14 cm right from the reference), and right-full position (4.27 cm right from the reference), respectively.

|  | **LF** | **LH** | **R0** | **RH** | **RF** |
| --- | --- | --- | --- | --- | --- |
| A (mm) | 557.8 ± 42.8 | 552.5 ± 44.5 | 548.3 ± 42.0 | 542.6 ± 43.0 | 537.5 ± 41.2 |
| B45 (mm) | 510.8 ± 38.6 | 506.3 ± 40.7 | 503.5 ± 37.7 | 498.1 ± 39.3 | 495.0 ± 37.1 |
| B90 (mm) | 477.9 ± 37.5 | 473.2 ± 40.1 | 471.4 ± 36.9 | 465.7 ± 38.8 | 462.5 ± 37.0 |
| B135 (mm) | 457.8 ± 38.1 | 453.3 ± 41.1 | 452.2 ± 38.1 | 446.3 ± 40.2 | 443.0 ± 38.4 |
| B180 (mm) | 448.9 ± 38.6 | 444.5 ± 41.6 | 443.7 ± 38.8 | 437.6 ± 41.1 | 434.6 ± 39.0 |
| B225 (mm) | 446.7 ± 39.2 | 442.9 ± 41.5 | 442.0 ± 39.2 | 435.9 ± 41.6 | 432.9 ± 39.3 |
| TP (mm) | 457.2 ± 38.9 | 453.5 ± 40.4 | 452.2 ± 38.7 | 446.3 ± 40.9 | 443.4 ± 38.1 |
| TC (mm) | 467.4 ± 39.2 | 464.4 ± 39.7 | 462.2 ± 38.5 | 456.6 ± 40.2 | 453.3 ± 37.3 |
| D225 (mm) | 519.0 ± 40.7 | 515.9 ± 39.9 | 511.5 ± 37.3 | 506.7 ± 38.5 | 502.6 ± 37.3 |
| D180 (mm) | 549.3 ± 40.7 | 546.4 ± 38.9 | 541.8 ± 36.0 | 536.5 ± 37.5 | 531.7 ± 36.4 |
| D135 (mm) | 577.5 ± 41.0 | 574.1 ± 39.3 | 569.7 ± 36.2 | 564.1 ± 37.3 | 558.6 ± 36.5 |
| D90 (mm) | 602.6 ± 42.1 | 599.7 ± 39.5 | 594.9 ± 36.4 | 589.2 ± 38.0 | 582.9 ± 36.8 |
| D45 (mm) | 621.3 ± 42.6 | 618.3 ± 40.3 | 614.0 ± 36.8 | 607.9 ± 38.0 | 601.4 ± 37.2 |
| I (mm) | 630.1 ± 43.0 | 627.3 ± 40.4 | 622.5 ± 36.9 | 616.6 ± 38.5 | 609.6 ± 37.7 |

**Table S17.** Anteroposterior centre of mass position (*COM_X_*) (mean ± SD) for 5 mediolateral ball positions over fourteen swing events analyzed: Address (A), backswing 45° (B45), backswing 90° (B90), backswing 135° (B135), backswing 180° (B180), backswing 225° (B225), transition of the pelvis (TP), transition of the club (TC), downswing 225° (D225), downswing 180° (D180), downswing 135° (D135), downswing 90° (D90), downswing 45° (D45), and impact (I). LF, LH, R0, RH, and RF stand for left-full position (4.27 cm left from the reference), left-half position (2.14 cm left from the reference), reference ball position (i.e. preferred ball position), right-half position (2.14 cm right from the reference), and right-full position (4.27 cm right from the reference), respectively.

|  | **LF** | **LH** | **R0** | **RH** | **RF** |
| --- | --- | --- | --- | --- | --- |
| A (mm) | 331.6 ± 26.1 | 331.7 ± 27.0 | 332.1 ± 26.6 | 333.2 ± 26.7 | 333.4 ± 26.7 |
| B45 (mm) | 329.4 ± 28.4 | 329.3 ± 29.0 | 329.7 ± 29.1 | 331.3 ± 28.6 | 330.9 ± 28.8 |
| B90 (mm) | 324.3 ± 29.4 | 323.8 ± 30.1 | 324.2 ± 29.9 | 325.9 ± 29.6 | 325.3 ± 29.6 |
| B135 (mm) | 317.3 ± 29.5 | 316.6 ± 30.1 | 317.3 ± 29.6 | 318.8 ± 29.6 | 318.0 ± 29.5 |
| B180 (mm) | 311.1 ± 29.5 | 310.4 ± 29.8 | 311.1 ± 29.3 | 312.4 ± 29.5 | 311.5 ± 29.3 |
| B225 (mm) | 304.3 ± 29.8 | 303.5 ± 29.7 | 304.3 ± 29.3 | 305.5 ± 29.6 | 304.6 ± 29.5 |
| TP (mm) | 299.7 ± 30.5 | 298.6 ± 30.9 | 299.5 ± 30.7 | 300.3 ± 31.0 | 299.4 ± 30.7 |
| TC (mm) | 301.1 ± 30.0 | 300.2 ± 30.4 | 301.0 ± 30.7 | 301.7 ± 30.7 | 301.0 ± 30.5 |
| D225 (mm) | 322.8 ± 27.1 | 322.5 ± 27.2 | 323.2 ± 27.6 | 324.5 ± 27.7 | 324.3 ± 27.5 |
| D180 (mm) | 329.1 ± 27.6 | 329.2 ± 28.0 | 330.3 ± 28.5 | 331.3 ± 28.5 | 331.3 ± 28.2 |
| D135 (mm) | 329.9 ± 27.6 | 330.1 ± 28.2 | 331.5 ± 28.9 | 332.7 ± 28.6 | 332.7 ± 28.4 |
| D90 (mm) | 327.1 ± 27.6 | 327.4 ± 28.4 | 329.2 ± 29.0 | 330.5 ± 28.6 | 330.5 ± 28.4 |
| D45 (mm) | 323.0 ± 27.7 | 323.3 ± 28.4 | 325.3 ± 29.1 | 326.6 ± 28.6 | 326.7 ± 28.5 |
| I (mm) | 320.5 ± 27.7 | 320.9 ± 28.4 | 323.1 ± 29.1 | 324.4 ± 28.5 | 324.6 ± 28.4 |
